# The Dual Subsurface Hydrogen (2H’) Mechanism for Ethylene Hydrogenation on Pd

**DOI:** 10.1021/acs.jpcc.5c02240

**Published:** 2025-08-07

**Authors:** Nicholas Golio, Andrew J Gellman

**Affiliations:** † Department of Chemical Engineering, Carnegie Mellon University, 5000 Forbes Ave, Pittsburgh, Pennsylvania 15213, United States; ‡ W.E. Scott Institute for Energy Innovation, Carnegie Mellon University, 5000 Forbes Ave, Pittsburgh, Pennsylvania 15213, United States

## Abstract

A microkinetic model for ethylene hydrogenation on Pd that includes the presence of subsurface hydrogen (H’) was developed by adapting the existing Horiuti–Polanyi framework. This reaction mechanism, known as the Dual Subsurface Hydrogen (2H’) mechanism, is an extension of a reaction model that was initially proposed to resolve inconsistencies in the Langmuir–Hinshelwood mechanism for the H_2_-D_2_ exchange reaction. The 2H’ mechanism accurately characterizes surface reactions on Pd-based alloy surfaces by accounting for the presence of H’ in the subsurface, which activates the adsorbed H atoms on the top surface, causing them to react. In this work, we derive a 2H’ mechanism for the hydrogenation of ethylene to ethane and compare the implications of the model to experimental results obtained on a Ag_*x*_Pd_1–*x*_ Composition Spread Alloy Film (CSAF). The ethylene hydrogenation reaction order in H_2_ predicted by the 2H’ mechanism, *n*_H2_, was consistent with *n*_H2_ = 0.69 ± 0.18 measured on Pd within the temperature range 345–405 K. In addition, the 2H’ rate law for ethane production was fit to experimental measurements of ethane production on Pd to estimate the effective hydrogenation rate constant, *k*_eff_, and the energy barriers for ethylene adsorption and desorption. Kinetic parameter estimation bounded the effective hydrogenation rate constant, *k*_eff_, to between 10^10^ and 10^14^ mol/m^2^/sec and predicted that the ethylene adsorption energy, $\Delta E_{ads}^{E}$ , is on the order of ∼10 kJ/mol. Development of the 2H’ mechanism for more complex reactions, like ethylene hydrogenation, shows the necessity for considering the presence of subsurface hydrogen in properly modeling surface reactions on Pd.

## Graphical abstract

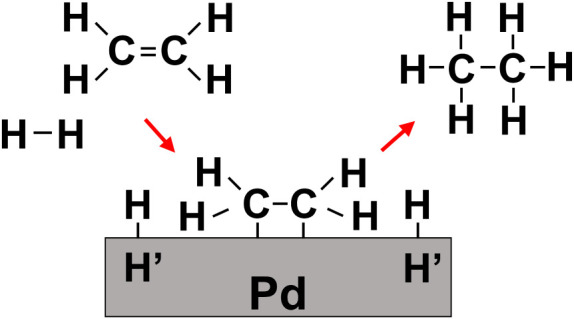

## 1 Introduction

Catalytic hydrogenation reactions performed on transition metal catalysts are critically important to industrial research and development. The hydrogenation of ethylene (C_2_H_4_) has been widely studied because it is the simplest alkene and can serve as an effective probe reaction for understanding the mechanism and kinetics of the hydrogenation of more complex olefins and aromatics.^1,2^ Ethylene hydrogenation is a relatively straightforward reaction to study because it occurs at room temperature and atmospheric pressure with fast turnover rates (∼10 site^–1^s^–1^).^3^ Ethylene hydrogenation has been characterized on several transition metals, with Pt and Pd being particularly active for the reaction.^4–11^ Experimental studies conducted on Pt and Pd have reported that the apparent activation energy for the production of ethane (C_2_H_6_) is between ∼33 kJ/mol and ∼46 kJ/mol;^1,3,9,12^ however, the energy barrier for each mechanistic step is not fully characterized.

There have been several investigations into the reaction mechanism for ethylene hydrogenation,^5,13–16^ which attempt to enumerate the elementary steps occurring on the surface. The most well-known mechanism for ethylene hydrogenation (Figure 1) was proposed by Horiuti and Polanyi in 1934, which considers adsorbed ethylene in its di-σ-bonded configuration and as a partially hydrogenated ethyl (C_2_H_5_) species.^17^ Since the proposal of the Horiuti–Polanyi mechanism, it has been discovered that ethylene can transform into several other surface intermediates, including π-bonded ethylene, ethylidyne (CCH_3_), and vinyl (CH=CH_2_),^3,14^ which make the surface chemistry much more complex than originally anticipated. While adsorbed ethylene can be converted into other surface species, not all intermediates contribute to the reaction pathway for ethylene hydrogenation. For example, it was observed that, when present, ethylidyne only exists as a spectator species and does not undergo direct conversion to ethane.^5,18,19^ However, it has been shown that under experimental conditions where the hydrogen pressure greatly exceeds the ethylene pressure, i.e., *P*_H2_ ≫ *P*_E_ , the surface is saturated with hydrogen atoms, making the formation of ethylidyne unlikely.^5,14^ In addition, it has been shown using Pt catalysts that both π-bonded and di-σ-bonded ethylene have reaction pathways that lead to the production of ethane.^18,20^ Consequently, the complex surface chemistry involved in the hydrogenation of adsorbed ethylene molecules can be somewhat simplified when kinetic analysis is performed under certain reaction conditions.

**Figure 1:**
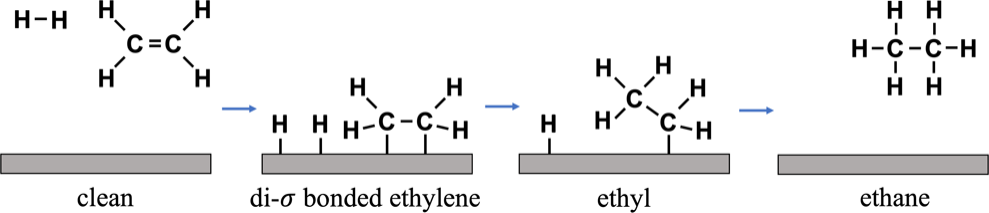
Schematic diagram of the Horiuti–Polanyi mechanism for ethylene hydrogenation. H_2_ adsorbs dissociatively, and ethylene adsorbs molecularly in a di-σ-bonded configuration, with each −CH_2_ group interacting with the surface. The reaction between adsorbed H atoms and adsorbed ethylene molecules occurs stepwise in two hydrogenation steps, after which the fully hydrogenated ethane molecule desorbs instantaneously into the gas phase.

The interaction of H_2_ with transition metal surfaces is fundamental to understanding the process by which ethylene hydrogenation occurs. Previously, we have proposed a reaction mechanism for the interaction of H_2_ with Pd and Ag_*x*_Pd_1–*x*_ alloy catalysts, known as the Dual Subsurface Hydrogen (2H’) mechanism. The 2H’ mechanism incorporates the effect of two subsurface hydrogen atoms, denoted as H’, in facilitating the adsorption and desorption of H_2_ occurring on the top surface.^21,22^ The 2H’ mechanism includes a surface-to-subsurface diffusion equilibrium constant, *K*_ss_, that describes the exchange of adsorbed surface H with absorbed subsurface H’. Once equilibrium is established between the surface and the subsurface, the 2H’ mechanism stipulates that two H’ are present in the immediate subsurface in order to activate surface H atoms for H_2_ adsorption and desorption.

The 2H’ mechanism was first applied to the H_2_-D_2_ exchange reaction on Pd in order to resolve inconsistencies between experimental measurements and the kinetic behavior predicted by the previous framework, the Langmuir–Hinshelwood (LH) mechanism. In particular, the H_2_-D_2_ exchange reaction order with respect to hydrogen predicted by the LH mechanism is $n_{H2}^{LH}=-1$ when *P*_H2_ ≫ *P*_D2_, which arises from the competition between H_2_ and D_2_ for adsorption sites on a nearly saturated surface, i.e., θ ≅ 1. However, experimental measurements by Sen et al. using a Ag_*x*_Pd_1–*x*_ Composition Spread Alloy Film (CSAF) showed that *n*_H2_ = 0 at all catalyst compositions when θ ≅ 1 and *P*_H2_ ≫ *P*_D2_. While *n*_H2_ = 0 is inconsistent with LH kinetics under these conditions, it aligns with the prediction obtained using the 2H’ mechanism for H_2_-D_2_ exchange.^21^ This non-LH result for the reaction order is supported by a similar study^23^ involving H_2_-D_2_ exchange on Pd (111) and Pd nanoparticles, where *n*_D2_ = 0 when θ ≅ 1 and *P*_D2_ ≫ *P*_H2_.

Subsequent to analyzing the reaction order with respect to H_2_, the rate law for H_2_-D_2_ exchange given by the LH and 2H’ mechanisms was fit to experimental measurements of HD production using the Ag_*x*_Pd_1–*x*_ CSAF at different reaction temperatures and inlet partial pressures of H_2_ and D_2_.^22,24^ These studies allowed us to extract estimates for the kinetic parameters describing the H_2_-D_2_ exchange reaction using different reaction mechanisms. Kinetic parameter fitting revealed another inconsistency in the LH mechanism, as it predicted that Pd operates in an adsorption-limited regime, with low surface coverage and a high energy barrier to H_2_ adsorption, $\Delta E_{ads-H2}^{‡}$ = 51.1 ± 0.6 kJ/mol.^22^ This prediction disagrees with numerous studies of H_2_ adsorption on Pd surfaces, which have shown that it adsorbs with a negligible barrier to dissociation and a high heat of adsorption.^25–27^ On the other hand, the 2H’ mechanism correctly predicted $\Delta E_{ads-H2}^{‡}$ = 0 kJ/mol and was also able to match density functional theory (DFT) predictions for the surface-to-subsurface diffusion energy, Δ*E*_ss_, of adsorbed H on Pd(111) and Pd(100).^27^ Fitting the rate law from the 2H’ mechanism to experimental measurements of H_2_-D_2_ exchange across Ag_*x*_Pd_1–*x*_ composition space yielded nearly identical kinetic parameters for all alloys with *x*_Pd_ ≥ 0.64, indicating that catalysts rich in Pd behave as if they were pure, bulk-like Pd.^24^ For Ag_*x*_Pd_1–*x*_ alloy compositions with *x*_Pd_ ≥ 0.64, the kinetic parameter ranges given by the 2H’ mechanism are $\Delta E_{ads-H2}^{‡}$ = 0–10 kJ/mol for dissociative H_2_ adsorption, $\Delta E_{des-H2}^{‡}$ = 30–65 kJ/mol for associative H_2_ desorption, and Δ*E*_ss_ = 20–35 kJ/mol for surface-to-subsurface H atom diffusion.^24^ The fact that the kinetic parameters for H_2_-D_2_ exchange vary minimally with respect to *x*_Pd_ at Pd-rich compositions suggests that the reaction only occurs on bulk-like Pd domains and that Ag merely serves as a diluent, restricting the total surface area available for reaction. This result is consistent with several studies that have predicted that H_2_ adsorption onto Ag single crystal surfaces is endothermic and does not occur spontaneously at room temperature.^28–31^ Thus, kinetic parameter estimation combined with the observed reaction order in H_2_ across Ag_*x*_Pd_1–*x*_ composition space provides strong evidence that the 2H’ mechanism is currently the most accurate model for describing H_2_-D_2_ exchange, and more broadly, for characterizing H_2_ adsorption and desorption onto/from Pd surfaces.

Similar to the improvements made to the Langmuir–Hinshelwood mechanism, the inclusion of subsurface hydrogen has the potential to improve the existing Horiuti–Polanyi framework. For nearly a century, the Horiuti–Polanyi mechanism has been the standard for describing hydrogenation reactions in heterogeneous catalysis. However, recent works using density functional theory (DFT) have shown a preference for a non-Horiuti–Polanyi mechanism in the hydrogenation of small-chain molecules, such as acrolein and acetylene.^32–35^ DFT calculations have shown that the non-Horiuti–Polanyi mechanism is favored on metallic surfaces having high energy barriers to H_2_ dissociation. For example, on inactive Au and Ag surfaces, the H atom adsorption energy is weak, leading to a preference for the molecular adsorption of H_2_ from the gas phase.^34^ When H_2_ is molecularly adsorbed onto catalyst surfaces, the reaction must proceed via a non-Horiuti–Polanyi mechanism since the stepwise addition of H atoms is no longer possible. However, on active catalysts with facile H_2_ dissociation, such as Pt and Pd, the classic Horiuti–Polanyi mechanism is still expected to be dominant. Unfortunately, these DFT studies only consider adsorbates on the top surface when calculating energy states for the proposed hydrogenation mechanisms and fail to incorporate the effect of subsurface hydrogen, H’, which is likely present just below the surface. The incorporation of H’ into these energy calculations is particularly important for Pd catalysts, as DFT studies have shown that the presence of H’ boosted acetylene hydrogenation activity on Pd (111), (100), and (211) by lowering the energy barrier for hydrogenation.^36,37^ Therefore, it is possible that accounting for the presence of H’ in calculating the adsorption and reaction energies of surface species might improve the consistency of the classic Horiuti–Polanyi mechanism. In other words, the non-Horiuti–Polanyi behavior observed by others might be more appropriately explained by the effect of subsurface species on the reaction rate and not by a change in the nature of H_2_ adsorption.

In this work, we extend the 2H’ framework established for H_2_-D_2_ exchange to obtain a microkinetic model for ethylene hydrogenation on Ag_*x*_Pd_1–*x*_ alloy catalysts that incorporates the presence of subsurface hydrogen, H’, into the reaction mechanism. The traditional Horiuti–Polanyi mechanism, shown in Figure 1, is simplified and adapted to preserve the dissociative adsorption of H_2_ and molecular adsorption of ethylene onto catalyst surfaces while incorporating the presence of subsurface H’, which is necessary to activate surface H atoms and facilitate the hydrogenation of ethylene molecules. Ultimately, we derive a rate law for ethylene hydrogenation using the 2H’ framework and compare the implications of the proposed mechanism with previous experimental measurements of ethylene hydrogenation taken across an Ag_*x*_Pd_1–*x*_ CSAF.^11^ In particular, the equation for the rate law derived from the 2H’ mechanism is used to obtain predictions for the reaction order with respect to H_2_, *n*_H2_ , under different experimental conditions. Comparison of these model-predicted values of *n*_H2_ show good agreement with the experimental measurements that were taken over an order-of-magnitude change in the H_2_ partial pressure. Additionally, the rate law obtained for ethane production, *r*_C2H6_, when applying the 2H’ framework is fit to the measured ethane production rate on the pure Pd catalyst (i.e., *x*_Pd_ = 1) to estimate the key kinetic parameters defining the reaction mechanism. Kinetic parameter fitting yields estimates for the energy barriers for ethylene adsorption, $\Delta E_{ads-E}^{‡}$ , ethylene desorption $\Delta E_{des-E}^{‡}$ , and the effective hydrogenation rate constant, *k*_eff_, by fixing the kinetic parameters for H_2_ adsorption, desorption, and surface-to-subsurface diffusion to the values previously determined from our study of H_2_-D_2_ exchange.^22,24^ The consistency of the 2H’ mechanism with experimental measurements of ethylene hydrogenation on Pd shows the potential for developing subsurface models to describe increasingly complex reactions.

## 2 Experimental Section

Note that the experimental data set for ethylene hydrogenation, collected using an Ag_*x*_Pd_1–*x*_ CSAF, comes from a previous publication whose primary objective was to compare the difference in ethylene conversion resulting from the presence or absence of a trace amount of O_2_ in the reactant feed.^11^ The presence of O_2_ drastically boosted the ethylene hydrogenation activity, and this effect was attributed to the creation of a restructured catalyst surface due to the simultaneous resegregation and activation of Pd atoms. In the absence of O_2_, equilibrium Ag_*x*_Pd_1–*x*_ catalyst surfaces were exposed, resulting in ethylene conversion at uniformly low conversion. In this work, only the data set collected without O_2_ present in the feed is used to evaluate the predictions of the 2H’ mechanism for ethylene hydrogenation, which are derived below. The choice for using the low conversion data set without O_2_ stems from the fact that the exact extent of the interaction between O_2_ and the restructured Ag_*x*_Pd_1–*x*_ catalyst surfaces has not been fully characterized, and therefore, it is unclear how the 2H’ mechanism should be applied in this case. Consequently, to adhere to the framework established for the 2H’ mechanism, we limit ourselves to reaction systems where only H_2_ and C_2_H_4_ molecules interact with the catalyst surface.

Detailed descriptions of CSAF preparation, CSAF characterization, and measurement of the ethylene hydrogenation activity using a multichannel microreactor array can be found in our previous publication.^11^ Below, we briefly summarize the Experimental sections pertinent to the collection of the high-throughput data set analyzed in this work.

### 2.1 CSAF Preparation

The Ag_*x*_Pd_1–*x*_ CSAF was prepared via simultaneous physical vapor deposition of Ag and Pd onto a 14 × 14 × 3 mm^3^ polished Mo substrate (Valley Design Corp.) using a rotatable shadow mask CSAF deposition tool that has been described previously.^38,39^ Independently controlled Ag and Pd electron beam evaporation sources with opposing flux gradients were used to deposit an ∼100 nm film of uniform thickness. After deposition, the CSAF was annealed at 800 K for 1 h in ultrahigh vacuum (UHV) to induce film crystallization,^40,41^ without resulting in alloy formation between the substrate and the constituent metals.^40–44^

### 2.2 Characterization of CSAF Composition

The bulk alloy composition and overall film thickness of the Ag_*x*_Pd_1–*x*_ CSAF were measured using energy-dispersive X-ray spectroscopy (EDX) in a Tescan VEGA3 scanning electron microscope equipped with an automated stage. The electron beam energy was set to 20 keV, and an EDX scan area of 50 × 50 μm^2^ was performed across a grid of 13 × 13 evenly spaced points spanning the 12 × 12 mm^2^ area at the center of the substrate. Quantification of the bulk alloy composition corresponding to each measurement site was performed using the Oxford Instruments INCA ThinFilmID software package, which accounted for the morphology of a thin Ag_*x*_Pd_1–*x*_ film deposited on a Mo substrate. EDX measurements across a grid of points at the center of the CSAF confirmed that the area spanned by the microreactor array contained all of binary composition space fairly uniformly, i.e., *x*_Pd_ = 0 → 1.^11^ The film thickness was determined by comparing the overall signal intensity at each point to that of a Ni reference material.

### 2.3 Measurement of Ethylene Hydrogenation Activity

The ethylene hydrogenation activity of the Ag_*x*_Pd_1–*x*_ CSAF was measured at 100 different alloy compositions using a high-throughput multichannel microreactor array, which has been described in detail elsewhere.^45^ Reactant mixtures of H_2_, ethylene (C_2_H_4_), and Ar were delivered continuously to 100 isolated regions on the Ag_*x*_Pd_1–*x*_ CSAF surface, and the reaction products were continuously withdrawn from each region for analysis using a Stanford Research Systems quadrupole mass spectrometer (RGA-200). The ethylene hydrogenation activity of the Ag_*x*_Pd_1–*x*_ catalysts contained on the CSAF was measured at atmospheric pressure (*P* ^tot^ = 760 Torr) with the ethylene partial pressure fixed at *P*_E_ = 25 Torr, over an H_2_ inlet partial pressure range spanning *P*_H2_ = 70–690 Torr, and a temperature range from *T* = 300–405 K in increments of 15 K.

The total reactant flow rate of 10 mL/min was split equally between the 100 channels of the microreactor array and two reference channels having 0% ethylene conversion and 100% ethylene conversion, respectively. The 0% conversion reference channel delivered the reactant gas mixture directly to the gas sampling system, while the 100% conversion reference channel sent the mixture to an independent reactor loaded with a high surface area of Pd wire that completely converted the ethylene to ethane. The extent of ethylene hydrogenation, ξ, in the microreactor channels was determined by linear interpolation of the mass spectrometer signal intensities at *m*/*z* = 29 and 30 amu (corresponding to the product ethane molecule) between the signal intensities measured inside the 0% and 100% conversion reference channels. The experimental data set was collected by keeping the reaction temperature constant and varying the H_2_ inlet partial pressure. Three consecutive scans through all 102 outlet channels were taken once the system had reached steady-state after changing the reaction conditions. The average ethylene conversion across the three scans is reported at all Ag_*x*_Pd_1–*x*_ compositions, reaction temperatures, and inlet hydrogen pressures, i.e., ξ­(*x*_Pd_,*T*,*P*_H2_).

## 3 Results

### 3.1 Ethylene Hydrogenation Activity across Ag_*x*_Pd_1‑*x*_ Composition Space

The entire data set showing the ethylene hydrogenation activity of the Ag_*x*_Pd_1–*x*_ CSAF as a function of alloy composition, reaction temperature, and inlet hydrogen pressure can be found in our previous publication.^11^ The ethylene hydrogenation activity of the Ag_*x*_Pd_1–*x*_ CSAF was measured by flowing H_2_, C_2_H_4_, and Ar mixtures into the microreactor at a constant reaction temperature, inlet pressure, and total flow rate, and measuring the product gas composition in the 100 outlet channels by mass spectrometry. In total, the extent of ethylene hydrogenation was obtained for 100 different Ag_*x*_Pd_1–*x*_ catalyst compositions spanning *x*_Pd_ = 0–1, at 8 different reaction temperatures from *T* = 300–405 K, and 5 different hydrogen partial pressures from $P_{H2}^{in}$ = 70–690 Torr, i.e., ξ­(x_Pd_,*T*,*P*_H2_).

Figure 2 shows a subset of the data set consisting of the extent of ethylene conversion (ξ) versus *x*_Pd_ and *T* when $P_{H2}^{in}$ = 690 Torr, which is the inlet hydrogen partial pressure with the highest overall activity. As shown in Figure 2, the ethylene hydrogenation activity of the Ag_*x*_Pd_1–*x*_ CSAF was generally low, with the maximum conversion of ξ ≈ 0.4 only being achieved on pure Pd (i.e., *x*_Pd_ = 1) at the highest reaction temperature, *T* = 405 K. As expected, the ethylene conversion decreases when decreasing both *x*_Pd_ and *T*. In fact, no ethylene hydrogenation activity was observed for alloys with *x*_Pd_ ≤ 0.9 at any *T* or *P*_H2_. This inactivity for *x*_Pd_ ≤ 0.9 is presumed to result from the saturation of Ag atoms on the top surface of the alloy. The segregation of Ag atoms from the bulk to the top surface minimizes the surface free energy of the system and presumably leaves no Pd domains of appreciable size accessible for catalysis, rendering the alloy inactive.^11^ This is due to the fact that the surface free energy^46^ of Ag(100) is only 0.71 J/m^2^, while that of Pd(100) is nearly twice as large at 1.37 J/m^2^. The significant difference in the surface free energies leads to predictions of Ag surface segregation in Ag/Pd(111) alloy slabs, resulting in inactive Ag-film covered surfaces.^47^ On the other hand, bulk alloy compositions with *x*_Pd_ > 0.9 appear to be sufficiently Pd-rich to ensure that there is enough Pd present on the top surface to convert ethylene to ethane, despite the fact that the surface is likely still Ag-enriched. From a kinetic modeling perspective, the ethylene hydrogenation data set with generally low conversion, i.e., ξ ≤ 0.4, is advantageous since it allows us to neglect the readsorption of ethane molecules from the gas phase onto the catalyst surface. In order to justify this simplification to the reaction mechanism, only those data points with less than 10% ethylene conversion (i.e., ξ < 0.1) were used in our analysis. As derived in Section 4, the rate law for ethane production resulting from this simplification allows us to evaluate the proposed 2H’ mechanism for ethylene hydrogenation using these experimental measurements at sufficiently low conversion.

**Figure 2:**
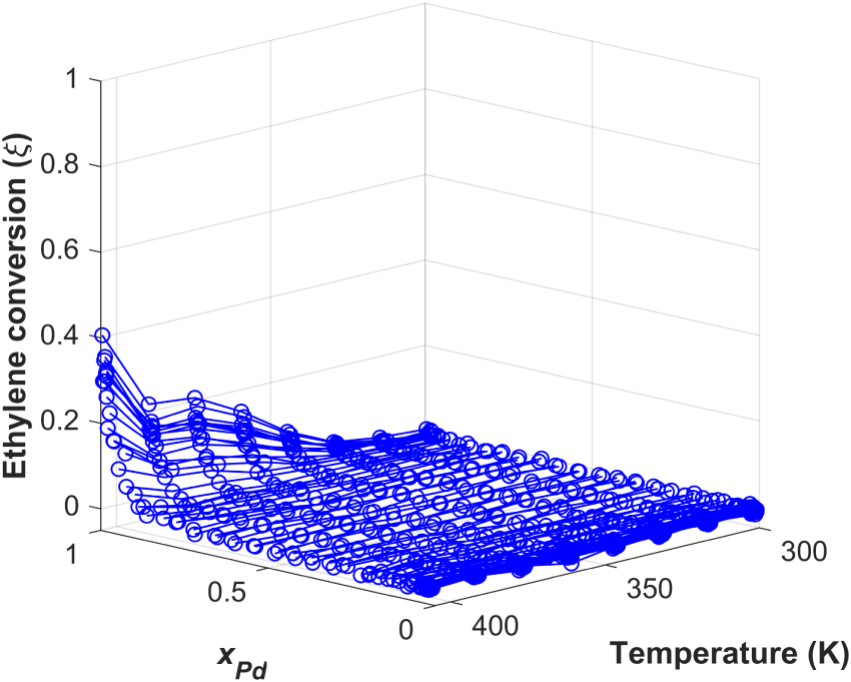
Extent of ethylene conversion, ξ , versus *x*_Pd_ and reaction temperature (K) at the inlet hydrogen partial pressure with the highest activity. The inlet partial pressures of the reactant stream were $P_{H2}^{in}$ = 690 Torr and $P_{E}^{in}$ = 25 Torr, with Ar composing the balance to achieve *P* ^tot^ = 760 Torr at a total flow rate of 10 mL/min. The maximum conversion of ξ ≈ 0.4 is achieved when *x*_Pd_ = 1 and *T* = 405 K. Decreasing *x*_Pd_ and *T* both result in decreases in ξ. No conversion is observed when *x*_Pd_ ≤ 0.9 or when *T* ≤ 330 K. The entire data set showing ξ versus *x*_Pd_ and *T* at all other values of $P_{H2}^{in}$ can be found in our previous publication.^11^ Figure adapted with permission from ref.^11^ Copyright 2023 American Chemical Society.

### 3.2 Ethylene Hydrogenation Reaction Order in H_2_

Using the ethylene hydrogenation data set^11^ (shown partially in Figure 2), we can estimate the reaction order with respect to hydrogen, *n*_H2_, (eq 1) since the measured ethylene conversion, ξ, is proportional to the total rate of ethane production, *r*_C2H6_, in the limit of low conversion (i.e., ξ < 0.1). Thus, finding the change in log­(ξ) with respect to log­(*P*_H2_) allows us to approximate n_H2_ for each alloy catalyst across the range of reaction temperatures.

1 $$n_{H2}=\frac{d\left(\log\left(r_{C2H6}\right)\right)}{d\left(\log\left(P_{H2}\right)\right)}\propto \frac{d\left(\log\left(\xi \right)\right)}{d\left(\log\left(P_{H2}\right)\right)}$$

Estimates of *n*_H2_ were determined by plotting log­(ξ) versus log­(*P*_H2_) for each alloy composition at all reaction temperatures and calculating the slope of the line of best fit. Figure S1 shows plots of log­(ξ) versus log­(*P*_H2_) for the 14 most Pd-rich catalysts on the Ag_*x*_Pd_1–*x*_ CSAF, ranging from *x*_Pd_ = 1–0.93, several of which have nominally identical bulk compositions. In Figure S1, all data points of the same color were measured at the same reaction temperature and are fitted by a line of best fit, the slope of which is *n*_H2_ shown at the right of each subplot. Note that Figure S1 contains all possible estimates for *n*_H2_ that can be obtained from this low conversion data set, since catalysts with *x*_Pd_ ≤ 0.9 were inactive for ethylene hydrogenation. Note also that some data points were excluded from Figure S1 either because the conversion was below the noise level, ξ < 0.02 (i.e., log­(ξ) <−1.7), or because there was insufficient data to fit a line (i.e., <3 data points).

An important observation from Figure S1 is that there is good agreement in *n*_H2_ for alloys with nominally identical bulk compositions. This indicates a high level of reproducibility and internal consistency of the data set. The value of *n*_H2_ predicted across all alloy compositions is positive, i.e., *n*_H2_ > 0 and appears to increase gradually as the reaction temperature increases. For example, the minimum value of *n*_H2_ = 0.33 occurs on Ag_0.05_Pd_0.95_ at 360 K, and the maximum value of *n*_H2_ = 1.09 occurs on Ag_0.03_Pd_0.97_ at 405 K. Since only Ag_*x*_Pd_1–*x*_ catalysts with *x*_Pd_ ≥ 0.93 displayed sufficient activity for ethylene hydrogenation, it can be assumed that Pd bears the entire catalytic load for the reaction and that Ag merely acts as a diluent. The same phenomenon was observed when performing H_2_-D_2_ exchange on an Ag_*x*_Pd_1–*x*_ CSAF similar to the one used in this work, in which the kinetic parameters predicted using the 2H’ mechanism were statistically indistinguishable from those predicted on pure Pd when *x*_Pd_ ≥ 0.64.^24^ Given that the energy barriers to H_2_ adsorption, desorption, and surface-to-subsurface diffusion were all equivalent for alloy compositions with *x*_Pd_ ≥ 0.64, this suggests that these Pd-rich alloys behave catalytically as if they are pure Pd. The lower surface free energy of Ag relative to Pd results in a high probability that Ag atoms are present on the top surface of the alloy even at dilute Ag bulk concentrations, and this is observed in the gradual decrease in activity as *x*_Pd_ decreases (Figure 2). However, our results suggest that the Pd domains remaining on the surface are large and continuous enough to behave as if they are pure, bulk-like Pd. Support for this claim comes from a related work studying the catalytic activity of Ag-rich Ag_*x*_Pd_1–*x*_ nanoparticles, which found that large clusters of Pd behaving like pure, bulk-like Pd were present and well-dispersed on the surface when *x*_Pd_ ≥ 0.33.^48^ Since this composition threshold is far below *x*_Pd_ ≥ 0.93 for the active Ag_*x*_Pd_1–*x*_ catalysts in this work, we expect that the changing bulk composition of the Ag_*x*_Pd_1–*x*_ CSAF only reflects the reduction in the total Pd surface area accessible for catalysis. Consequently, the estimates of *n*_H2_ can be averaged across alloy composition to obtain the average ethylene hydrogenation reaction order on Pd at different reaction temperatures.

Figure 3 shows the ethylene hydrogenation reaction order in H_2_, *n*_H2_ , averaged over Ag_*x*_Pd_1–*x*_ catalyst compositions with *x*_Pd_ ≥ 0.93 (shown in Figure S1) versus the reaction temperature, *T*. A slight dependence on *T* is observed, as the average *n*_H2_ increases from 0.53 at 345 K to 0.91 at 405 K. The red line in Figure 3 describes the relationship between *n*_H2_ and *T* , the slope of which is $\frac{dn_{H2}}{dT}$ = 0.006 and characterizes the increase in *n*_H2_ per K increase in the reaction temperature. Since the error bars in Figure 3 (representing one standard deviation from the mean value of *n*_H2_) are almost all overlapping, it can be concluded that *n*_H2_ has a real, but weak *T* dependence over the range of reaction temperatures used in this study. The most useful information to note from Figure 3 is that the H_2_ reaction order for ethylene hydrogenation on Pd is a positive value between $\frac{1}{2}$ and 1 (i.e., $\frac{1}{2}\leq n_{H2}\leq 1)$ across this range of reaction temperatures. Limitations of the 2H’ mechanism prevent us from distinguishing between minor changes in n_H2_ with respect to *T* (as discussed further in Section 4.2). Therefore, we take the average reaction order over all temperatures, *n*_H2_ = 0.69 ± 0.18, to use for comparison with the predictions of the 2H’ mechanism that are derived in the following section. This value of *n*_H2_ = 0.69 ± 0.18 within the range *T* = 345–405 K represents the global average obtained from the experimental data set, with the understanding that ethylene hydrogenation only occurs on large bulk-like Pd domains and acknowledging the limitation that only broad trends in *n*_H2_ can be evaluated.

**Figure 3:**
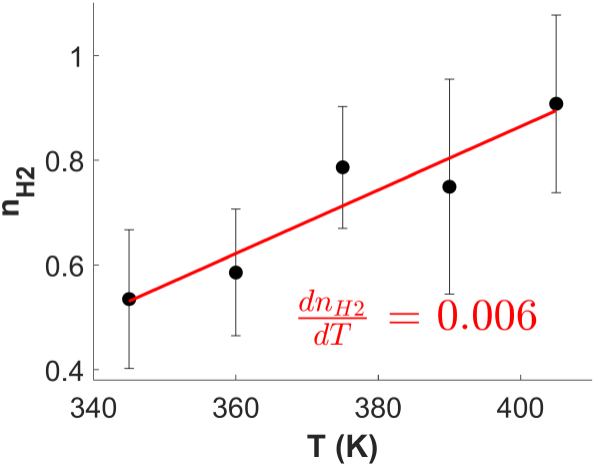
Average ethylene hydrogenation reaction order in H_2_, *n*_H2_ , over all Ag_*x*_Pd_1–*x*_ catalyst compositions shown in Figure S1 versus the reaction temperature, *T* , with error bars showing the standard deviation. Averaging over all catalyst compositions with *x*_Pd_ ≥ 0.93 is justified by the fact that Pd is expected to bear the entire catalytic load for ethylene hydrogenation and that Ag merely serves as a diluent. A slight dependence on *T* is observed as the average *n*_H2_ increases from 0.53 at 345 K to 0.91 at 405 K. The red line of best fit describes the relationship between *n*_H2_ and *T*, the slope of which is $\frac{dn_{H2}}{dT}$ = 0.006, characterizing the increase in $n_{H2}$ per K increase in the reaction temperature. Since *n*_H2_ only weakly depends on *T* within this range, an average over all reaction temperatures yields *n*_H2_ = 0.69 ± 0.18, which represents the global average for ethylene hydrogenation on Pd in the range *T* = 345–405 K.

## 4 Discussion

### 4.1 2H’ Mechanism for Ethylene Hydrogenation

In this section, we derive a microkinetic model for ethylene hydrogenation by extending the framework of the Dual Subsurface Hydrogen (2H’) mechanism that was originally applied to the H_2_-D_2_ exchange reaction. In doing so, we preserve the key elements of the Horiuti–Polanyi mechanism for ethylene hydrogenation, as shown in Figure 1, while simplifying the two stepwise hydrogenation steps into one simultaneous hydrogenation step, followed by instantaneous ethane desorption.

Figure 4 shows a schematic of the proposed 2H’ mechanism for ethylene hydrogenation. The surface and subsurface of the catalyst are divided into discrete adsorption sites that can be populated with either zero or one species per site. In Figure 4, the dissociative adsorption of H_2_ molecules from the gas phase into independent H atoms populates two adjacent surface sites. Similarly, the molecular adsorption of ethylene from the gas phase occupies two adjacent surface sites in order to preserve the di-σ-bonded configuration proposed by the Horiuti–Polanyi mechanism. This can be understood by considering that each half of the adsorbed ethylene molecule (E) in Figure 4 represents the interaction of a −CH_2_ group with the surface. It is important to note that H_2_ and ethylene adsorption are in competition with one another in this interpretation of the mechanism. While both competitive and noncompetitive adsorption pathways are possible for ethylene hydrogenation, the low ethylene pressure (i.e., $P_{E}^{in}$ = 25 Torr) and high reaction temperatures (i.e., *T* ≥ 290 K) used in our experiments are more consistent with a competitive adsorption model.^4,5^ To incorporate subsurface hydrogen, H’, into the reaction mechanism, adsorbed surface H can populate vacant subsurface sites through the process of surface-to-subsurface diffusion. The balance between adsorbed surface H and absorbed subsurface H’ is governed by the equilibrium constant, *K*_ss_, which is determined by taking the ratio of the diffusion rates of H atoms into and out of the subsurface.

**Figure 4:**
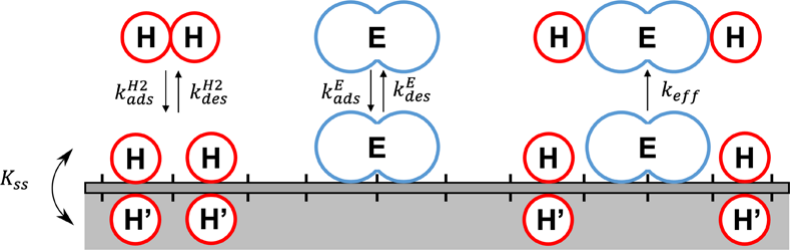
Schematic diagram of the proposed 2H’ mechanism for ethylene hydrogenation. H_2_ and ethylene (E) adsorb competitively on the catalyst surface into two adjacent empty sites. The equilibrium constant $K_{H2}=k_{ads}^{H2}/k_{des}^{H2}$ describes the dissociative adsorption and associative desorption of H_2_, and the equilibrium constant $K_{E}=k_{ads}^{E}/k_{des}^{E}$ describes the molecular adsorption and desorption of ethylene. The surface-to-subsurface diffusion equilibrium constant, $K_{ss}=k_{ss}^{in}/k_{ss}^{out}$ , describes the exchange of adsorbed H atoms on the top surface with absorbed H’ in the immediate subsurface. The presence of H’ in the subsurface facilitates both the adsorption and desorption of H_2_ and the hydrogenation of adsorbed ethylene. The hydrogenation of ethylene is represented as a single step with an effective hydrogenation rate constant, *k*_eff_. The reaction occurs when two adsorbed H atoms are influenced by two subsurface H’ in the vicinity of an E molecule and combine to form ethane (HEH), which desorbs instantaneously. In the limit of low conversion, the readsorption of ethane on the surface is negligible.

From the 2H’ mechanism established for H_2_-D_2_ exchange, H_2_ adsorption and desorption on the top surface are facilitated by the presence of two H’ atoms in the immediate subsurface. The presence of H’ can also influence the hydrogenation of adsorbed ethylene by interacting with surface H in the vicinity of adsorbed E, causing them to react. Similar to H_2_ adsorption and desorption, this mechanism for ethylene hydrogenation requires the simultaneous interaction of two subsurface H’ with two surface H to fully hydrogenate the ethylene molecule to ethane (HEH in Figure 4), which desorbs instantaneously. In this case, however, the two H’ need not be adjacent (as was assumed for H_2_ adsorption and desorption) as long as they are present in subsurface sites in the vicinity of each surface H participating in hydrogenation. It is worthwhile to mention that there is likely a range of interactions between the surface and the subsurface that extend beyond the site immediately below a given surface species. The most important factor is that there is one H’ present for every surface H participating in the reaction and that the species are close enough to create strong interactions between the two layers. Note that, in this way, the proposed 2H’ mechanism for ethylene hydrogenation preserves the influence of H’ on H, which was shown to be essential for accurately modeling the H_2_-D_2_ exchange reaction.^21^ In summary, all transformations of surface H, including adsorption, desorption, and reaction through the hydrogenation of ethylene, require activation from the presence of subsurface H’. While it is also possible that the presence of H’ has the potential to influence the adsorption and desorption of ethylene molecules, we assume that the effect is much less significant than for H due to the fact that the size of an ethylene molecule (E) is ∼4 times greater than that of an H atom.

In the schematic of the 2H’ mechanism for ethylene hydrogenation given in Figure 4, H_2_ and ethylene (E) adsorb competitively onto the catalyst surface. The equilibrium constant $K_{H2}=k_{\mathrm{ads}}^{H2}/k_{\mathrm{des}}^{H2}$ is the ratio between the rate constant for dissociative H_2_ adsorption, $k_{\mathrm{ads}}^{H2}$ , and the rate constant for associative H_2_ desorption, $k_{\mathrm{des}}^{H2}$ , under the influence of 2H’. The surface-to-subsurface diffusion equilibrium constant $K_{\mathrm{ss}}=k_{\mathrm{ss}}^{\mathrm{in}}/k_{ss}^{\mathrm{out}}$ is the ratio between the rate constant for H absorption into the subsurface, $k_{\mathrm{ss}}^{in}$ , and the rate constant for H’ reemergence on the top surface, $k_{ss}^{\mathrm{out}}$ . The equilibrium constant $K_{E}=k_{\mathrm{ads}}^{E}/k_{\mathrm{des}}^{E}$ is the ratio between the rate constant for molecular E adsorption, $k_{\mathrm{ads}}^{E}$ , and the rate constant for molecular E desorption, $k_{\mathrm{des}}^{E}$ . The effective ethylene hydrogenation rate constant is given by *k*_eff_, which incorporates the two stepwise hydrogenation steps shown in Figure 1 into a single step that results in the instantaneous desorption of ethane (HEH) from the surface. We define the variables θ_H_, θ_E_, and θ^’^_H_ to be the coverage of H on the surface, the coverage of E on the surface, and the coverage of H’ in the subsurface, respectively. At the steady-state reaction conditions experienced inside the microreactor, the change in the coverage of each species, *i*, with respect to time, is zero, i.e., $\frac{d\theta_{i}}{dt}=0$ . The steady-state equations describing θ_H_, θ_E_, and θ^’^_H_ for the 2H’ mechanism are given by eqs 2–4.

2 $$\frac{d\theta_{H}}{dt}=0=2k_{\mathrm{ads}}^{H2}P_{H2}{\left(1-\theta_{H}-\frac{1}{2}\theta_{E}\right)}^{2}\theta_{H}^{\prime 2}-2k_{\mathrm{des}}^{H2}\theta_{H}^{2}\theta_{H}^{\prime 2}-k_{ss}^{in}\theta_{H}\left(1-\theta_{H}^{\prime }\right)+k_{ss}^{\mathrm{out}}\theta_{H}^{\prime }\left(1-\theta_{H}-\frac{1}{2}\theta_{E}\right)$$

3 $$\frac{d\theta_{E}}{dt}=0=k_{\mathrm{ads}}^{E}P_{E}\left(1-\theta_{H}-\frac{1}{2}\theta_{E}\right)-\frac{1}{2}k_{\mathrm{des}}^{E}\theta_{E}$$

4 $$\frac{d\theta_{H}^{\prime }}{dt}=0=k_{ss}^{in}\theta_{H}\left(1-\theta_{H}^{\prime }\right)-k_{ss}^{\mathrm{out}}\theta_{H}^{\prime }\left(1-\theta_{H}-\frac{1}{2}\theta_{E}\right)$$

Under conditions of low ethylene conversion (i.e., ξ < 0.1), the pressure of ethane in the gas phase is negligible, i.e., *P*_C2H6_ ≈ 0, and ethane readsorption onto the surface can be ignored, resulting in an ethane production rate given by eq 5

5 $$r_{C2H6}=\frac{1}{2}k_{eff}\theta_{E}\theta_{H}^{2}\theta_{H}^{\prime 2}$$

The coverage terms in the rate expression can be calculated using the steady-state mass balance, eqs 2–4. A full solution to the 2H’ mechanism for ethylene hydrogenation is shown in the Supporting Information, including explicit derivation of the expressions for θ_H_, θ_E_, and θ^’^_H_ in terms of equilibrium constants and reactant partial pressures. Substitution of these coverage terms into eq 5 yields the ethane production rate, *r*_C2H6_, in eq 6 in terms of experimental parameters (*P*_H2_ and *P*_E_) and rate and equilibrium constants (*K*_H2_,*K*_E_,*K*_ss_, and *k*_eff_ ).

6 $$r_{C2H6}=\frac{k_{eff}K_{ss}^{2}K_{E}P_{E}K_{H2}^{2}P_{H2}^{2}}{{\left(1+\sqrt{K_{H2}P_{H2}}+K_{E}P_{E}\right)}^{3}{\left(1+K_{ss}\sqrt{K_{H2}P_{H2}}\right)}^{2}}$$

Eq 6 shows the rate of ethane production using the 2H’ mechanism for ethylene hydrogenation. It is important to note that the effective hydrogenation rate constant, *k*_eff_, incorporates both hydrogenation steps and the molecular desorption of ethane into a single step, which is an oversimplification of the actual hydrogenation process. Nonetheless, the rate law in eq 6 can be understood to represent the rate-limiting step for ethylene hydrogenation, which, for Pd surfaces^49–51^ is often believed to be the addition of the first H atom to adsorbed ethylene to create the ethyl intermediate shown in Figure 1. Thus, the implicit assumption is that the other steps in the reaction, namely the addition of the second H atom and the desorption of ethane, occur very fast relative to the addition of the first H and are thus not as kinetically relevant.

### 4.2 Reaction Order in H_2_ Given by the 2H’ Mechanism for Ethylene Hydrogenation

The reaction order with respect to H_2_, *n*_H2_, describes the dependence of the ethane production rate in eq 6 on the hydrogen partial pressure, *P*_H2_ . While *P*_H2_ appears in both the numerator and the denominator of eq 6, the expression can be simplified based on which terms in the denominator are expected to be dominant. Each term in the denominator in eq 6, ${\left(1+\sqrt{K_{H2}P_{H2}}+K_{E}P_{E}\right)}^{3}$ and ${\left(1+K_{ss}\sqrt{K_{H2}P_{H2}}\right)}^{2}$ , can be analyzed based on the relative magnitude of the quantities within the parentheses, which are determined by the product of kinetic parameters (i.e., equilibrium constants) and experimental conditions (i.e., reactant partial pressures). For example, under conditions where $\sqrt{K_{H2}P_{H2}}/\left(1+K_{E}P_{E}\right)\ll 1$ (i.e., $\sqrt{K_{H2}P_{H2}}\ll \left(1+K_{E}P_{E}\right))$ and $K_{ss}\sqrt{K_{H2}P_{H2}}\ll 1$ , both terms containing $P_{H2}$ in the denominator of eq 6 are negligible, and the dependence of $r_{C2H6}$ on $P_{H2}$ simplifies to $r_{C2H6}∼P_{H2}^{2}$ , which results in *n*_H2_ = 2. It is important to note that obtaining a prediction for $n_{H2}$ by analyzing the rate expression at different reaction conditions carries implicit assumptions about the surface H coverage (θ_H_) and subsurface H’ coverage (θ^’^_H_). For example, when $K_{ss}\sqrt{K_{H2}P_{H2}}\ll 1$ , it implies that the rate of adsorbed H diffusion into the subsurface is much slower than the rate of H’ resurfacing on the top surface, leading to a subsurface that is nearly vacant, θ^’^_H_ ≈ 0. Similarly, when $\sqrt{K_{H2}P_{H2}}/\left(1+K_{E}P_{E}\right)\ll 1$ , it implies that the rate of H_2_ adsorption on the surface is much slower than either the rate of H_2_ desorption and/or the adsorption equilibrium established for ethylene, resulting in a top surface that is H-depleted, θ_H_ ≈ 0. Full analysis of the rate expression under different reaction conditions and their corresponding implications for *n*_H2_ can be found in the Supporting Information. Table 1 presents a summary of different reaction conditions influencing the ethane production rate, their corresponding θ_H_ and θ^’^_H_ , and their prediction for the ethylene hydrogenation reaction order with respect to H_2_, *n*_H2_ .

**Table 1:** Ethylene Hydrogenation Reaction Orders in H_2_, *n*_H2_, Predicted by the 2H’ Mechanism at Different Reaction Conditions

Reaction Conditions				
$K_{ss}\sqrt{K_{H2}P_{H2}}$	≫ 1	≪ 1	≫ 1	≪ 1
$\sqrt{K_{H2}P_{H2}}/\left(1+K_{E}P_{E}\right)$	≫ 1	≫ 1	≪ 1	≪ 1

Coverages				
θ^’^_H_	≅ 1	≅ 0	≅ 1	≅ 0
θ_H_	≅ 2	≅ 1	≅ 0	≅ 0

Reaction Order in *P*_H2_				
*n*_H2_	$-\frac{1}{2}$	$\frac{1}{2}$	1	2

Table 1 shows the predictions for *n*_H2_ that can be obtained from the 2H’ mechanism for ethylene hydrogenation at the extremes of H and H’ coverage. These predictions can be compared with the experimentally measured values of *n*_H2_ that were obtained by performing ethylene hydrogenation on the Ag_*x*_Pd_1–*x*_ CSAF using the high-throughput microreactor array. From Figure 3, the average experimental *n*_H2_ for ethylene hydrogenation on Pd varied from 0.53 at 345 K to 0.91 at 405 K, with a global average of *n*_H2_ = 0.69 ± 0.18. These measurements of *n*_H2_ are consistent with the middle columns of Table 1, predicting either *n*_H2_ = $\frac{1}{2}$ when θ_H_ ≅ 1 and θ^’^_H_≅ 0 or *n*_H2_ = 1 when θ_H_ ≅ 0 and θ^'^_H_ ≅ 1 . It is important to note that the 2H’ mechanism is unable to distinguish between these two scenarios *a priori* due to the form of the rate equation, in which interchanging the values of $\theta_{H}^{2}$ and $\theta_{H}^{\prime 2}$ in eq 5 yields the same value of *r*_C2H6_. This is a known limitation of the 2H’ mechanism, which we reported previously for H_2_-D_2_ exchange.^22^ In essence, the 2H’ mechanism is unable to distinguish between surface and subsurface sites due to their equivalence with respect to H atoms. Thus, despite the fact that the experimental values of *n*_H2_ lie between two predictions of the 2H’ mechanism, only one set of conditions can be applied to our system, while the other is merely a symmetric solution resulting from swapping the surface and subsurface layers. In this case, it is necessary to use other predictions of the 2H’ mechanism and the adsorption behavior of H_2_ and ethylene in order to determine whether H atom saturation occurs in the surface or in the subsurface, while the other layer is nearly vacant.

In analyzing which set of reaction conditions and coverages is more consistent with our experiments, it is important to remember that ethylene hydrogenation was performed under conditions where *P*_H2_ ranged from 70 to 690 Torr while *P_E_* remained fixed at 25 Torr. In other words, *P*_H2_ ≫ *P*_E_ at all experimental conditions, and thus we expect the surface coverage of H to be dominant over E, i.e., θ_H_ ≫ θ_E_ . Taking this into account, the scenario where θ_H_ ≅ 1, corresponding to *n*_H2_ = $\frac{1}{2}$ , is much more probable than the scenario where θ_H_ ≅ 0, corresponding to *n*_H2_ = 1. Furthermore, the coverages θ_H_ ≅ 1 and θ^’^_H_ ≅ 0 predicted when *n*_H2_ = $\frac{1}{2}$ are also consistent with the surface and subsurface coverages that were calculated when applying the 2H’ mechanism to the H_2_-D_2_ exchange reaction.^22^ For H_2_-D_2_ exchange, the 2H’ mechanism’s prediction of *n*_H2_ = 0 consistent with experimental measurements^21^ when *P*_H2_ ≫ *P*_D2_ requires a nearly saturated top surface, θ ≅ 1, and a vacant subsurface, θ^*’*^ ≅ 0. It is reasonable to expect that the surface H and subsurface H’ coverages predicted by the 2H’ mechanism would be similar for H_2_-D_2_ exchange and for ethylene hydrogenation on Pd, especially due to the high H_2_ pressure used in both experiments. Therefore, the 2H’ prediction of $n_{H2}=\frac{1}{2}$ with θ_H_≅ 1 and θ^’^_H_ ≅ 0 is most strongly supported by the experimental data.

### 4.3 Fitting the 2H’ Rate Law to the Measured Ethane Production Rate on Pd

Beyond analysis of *n*_H2_, the 2H’ mechanism for ethylene hydrogenation can be evaluated by fitting the ethane production rate in eq 6 to the experimental data set collected using the Ag_*x*_Pd_1*x*_ CSAF^11^ to obtain estimates for the kinetic parameters defining the reaction mechanism. Measurements of the fractional ethylene conversion, ξ, can be converted to a molar flow rate of ethane exiting the microreactor, $F_{C2H6}^{\exp}$ , using the inlet volumetric flow rate of ethylene, *V̇* (m^3^/s), and the molar volume of an ideal gas (eq 7).

7 $$F_{C2H6}^{\exp}=\frac{\xi \mathrm{V̇}}{22.4\times 10^{-3}m^{3}/\mathrm{mol}}$$

It is important to note that there is a slight reduction in the total flow rate as ξ increases due to the consumption of two moles of reactants for every mole of ethane produced (i.e., H_2_ + C_2_H_4_ → C_2_H_6_). However, due to the low partial pressure of ethylene (*P*_E_ = 25 Torr) with respect to the total (P^tot^ = 760 Torr), the maximum reduction in the flow rate is only ∼3% when ξ = 1. Thus, the reduction in the flow rate is negligible, especially since the data set used for the fitting was collected in the low conversion regime (i.e., ξ < 0.1).

The ethane production rate, *r*_C2H6_ (mol/m^2^/s), predicted by the 2H’ mechanism in eq 6 can be converted to a model-predicted molar flow rate, $F_{C2H6}^{\mathrm{model}}$ (mol/s), since the catalyst surface area is defined by the dimensions of the gasket holes (700 × 800 μm^2^) that divide the surface of the CSAF into independent reaction volumes. The ethane flow rate predicted by the 2H’ model, therefore, is given by eq 8, where *A* represents the exposed catalyst surface area of 5.6 × 10^–7^ m^2^.

8 $$F_{C2H6}^{\mathrm{model}}=Ar_{C2H6}=\frac{Ak_{\mathrm{eff}}K_{ss}^{2}K_{E}P_{E}K_{H2}^{2}P_{H2}^{2}}{{\left(1+\sqrt{K_{H2}P_{H2}}+K_{E}P_{E}\right)}^{3}{\left(1+K_{ss}\sqrt{K_{H2}P_{H2}}\right)}^{2}}$$

The unknown kinetic parameters in the reaction mechanism for ethylene hydrogenation can be found by fitting the analytic expression for the ethane flow rate predicted by the 2H’ mechanism, $F_{C2H6}^{\mathrm{model}}$ , to the measured ethane flow rate, $F_{C2H6}^{\exp}$ , using an optimization routine, as was done previously in our study of H_2_-D_2_ exchange.^22,24^ This optimization routine involves minimizing the relative sum of squared errors, *χ* ^2^, between $F_{C2H6}^{\mathrm{model}}$ and $F_{C2H6}^{\exp}$ (eq 9) by varying the unknown kinetic parameters in $F_{C2H6}^{\mathrm{model}}$ over a well-defined search space.

9 $$\chi^{2}=\sum {\left(\frac{F_{C2H6}^{\mathrm{model}}-F_{C2H6}^{\exp}}{F_{C2H6}^{\exp}}\right)}^{2}$$

To properly define the kinetic parameter estimation problem, one must first evaluate the analytic expression for $F_{C2H6}^{\mathrm{model}}$ and reduce the degrees of freedom by determining all of the known quantities. For example, the inlet partial pressures of H_2_ and ethylene, *P*_H2_ and *P*_E_ , respectively, are known based upon the experimental conditions with which the data set was collected. In addition, the equilibrium constants for H_2_ adsorption $\left(K_{H2}\right)$ and surface-to-subsurface H diffusion (*K*_ss_) can be taken from our previous study of H_2_-D_2_ exchange on Pd using a similar Ag_*x*_Pd_1–*x*_ CSAF.^22,24^ The 2H’ mechanism for H_2_-D_2_ exchange predicted that the energy barrier for dissociative H_2_ adsorption is $\Delta E_{\mathrm{ads}-H2}^{‡}$ = 0 kJ/mol, the energy barrier for associative H_2_ desorption is $\Delta E_{\mathrm{des}-H2}^{‡}$ = 43 kJ/mol, and the surface-to-subsurface diffusion energy is Δ*E*_ss_ = 25 kJ/mol for pure Pd catalysts.^22^ These fitted parameters, along with their pre-exponential factors obtained from transition state theory,^16^ $v_{\mathrm{ads}}^{H2}$ = 10^2^ mol/m^2^/s/Torr, $v_{\mathrm{des}}^{H2}$ = 10^6^ mol/m^2^/s, and $v_{ss}^{H2}$ = 10^0^, are sufficient to determine *K*_H*2*_ and *K*_ss_ using eqs 10 and 11.

10 $$K_{H2}=\frac{k_{\mathrm{ads}}^{H2}}{k_{\mathrm{des}}^{H2}}=\frac{v_{\mathrm{ads}}^{H2}\exp\left(-\frac{\Delta E_{\mathrm{ads}-H2}^{‡}}{RT}\right)}{v_{\mathrm{des}}^{H2}\exp\left(\frac{-\Delta E_{\mathrm{des}-H2}^{‡}}{RT}\right)}$$

11 $$K_{ss}=v_{ss}\exp\left(\frac{-\Delta E_{ss}}{RT}\right)$$

12 $$K_{E}=\frac{k_{\mathrm{ads}}^{E}}{k_{\mathrm{des}}^{E}}=\frac{v_{\mathrm{ads}}^{E}\exp\left(-\frac{\Delta E_{\mathrm{ads}-E}^{‡}}{RT}\right)}{v_{\mathrm{des}}^{E}\exp\left(-\frac{\Delta E_{\mathrm{des}-E}^{‡}}{RT}\right)}$$

With *P*_H2_, *P*_E_, *K*_H2_, and *K*_ss_ known, the only unknown variables in eq 8 are the effective hydrogenation rate constant, *k*_eff_, and the ethylene adsorption equilibrium constant, *K*_E_. As shown in eq 12, while there are 4 kinetic parameters $(v_{\mathrm{ads}}^{E}$ , $v_{\mathrm{des}}^{E}$ , $\Delta E_{\mathrm{ads}-E}^{‡}$ , $\Delta E_{\mathrm{des}-E}^{‡})$ embedded in *K*_E_ (as for *K*_H2_), the pre-exponential factors for ethylene adsorption and desorption can be estimated using transition state theory. Transition state theory calculations for molecular adsorption and desorption give $v_{\mathrm{ads}}^{E}$ = 10^2^ mol/m^2^/s/Torr and $v_{\mathrm{des}}^{E}$ = 10^14^ mol/m^2^/s, respectively.^16^ Fixing the pre-exponents for ethylene adsorption and desorption results in only 3 unknown kinetic parameters in the model-predicted ethane production rate: *k*_eff_, $\Delta E_{\mathrm{ads}-E}^{‡}$ , and $\Delta E_{\mathrm{des}-E}^{‡}$ . Using eq 9, an optimization routine can now be implemented to fit *k*_eff_, $\Delta E_{\mathrm{ads}-E}^{‡}$ , and $\Delta E_{\mathrm{des}-E}^{‡}$ to the experimental data set collected using the Ag_*x*_Pd_1–*x*_ CSAF.^11^

To simplify our application of the 2H’ mechanism to the experimental data set, we limit our estimation of kinetic parameters to the ethane production of the pure Pd catalyst on the Ag_*x*_Pd_1–*x*_ CSAF, i.e., *x*_Pd_ = 1. In this way, we can neglect the effects of alloying Ag with Pd, even though our prior investigation of H_2_-D_2_ exchange on a similar Ag_*x*_Pd_1–*x*_ CSAF suggests that H_2_ is likely only activated through interactions with Pd for alloys with *x*_Pd_ ≥ 0.64.^24^ Using the single catalyst composition *x*_Pd_ = 1, the size of the experimental data set for ethylene hydrogenation consists of 40 data points comprising 8 reaction temperatures from *T* = 300–405 K and 5 inlet hydrogen pressures from $P_{H2}^{in}$ = 70–690 Torr. However, using only the data with ξ < 0.1 reduces the size of the data set used in the fitting to 28 points. The model-predicted ethane production rate, $F_{C2H6}^{\mathrm{model}}$ , was fit to $F_{C2H6}^{\exp}$ for the Pd catalyst with eq 9 using the MATLAB minimization tool *fmincon*. The optimization was performed by generating 500 initial guesses for the ethylene adsorption energy barrier $\left(\Delta E_{\mathrm{ads}-E}^{‡}\right)$ , the ethylene desorption energy barrier $\left(\Delta E_{\mathrm{des}-E}^{‡}\right)$ , and the effective hydrogenation rate constant (*k*_eff_), and then varying the parameters within their respective search space until the error between $F_{C2H6}^{\exp}$ and $F_{C2H6}^{\mathrm{model}}$ was minimized. The parameter space for $\Delta E_{\mathrm{ads}-E}^{‡}$ and $\Delta E_{\mathrm{des}-E}^{‡}$ was constrained between 0 and 100 kJ/mol, while the search space for *k*_eff_ ranged from 10^–20^ to 10^20^ mol/m^2^/s/Torr. The set of parameter values for $\Delta E_{\mathrm{ads}-E}^{‡}$ , $\Delta E_{\mathrm{des}-E}^{‡}$ , and *k*_eff_ that yielded the lowest value of χ^2^ among the 500 optimizations was chosen as the optimal solution.

The optimal fit obtained for the Pd catalyst is shown in Figure 5, which plots *F*_C2H6_ versus *T* at each value of $P_{H2}^{in}$ . The data points showing the experimental measurements of the ethane production rate, $F_{C2H6}^{\exp}$ , are fitted with curves of the same color showing the model-predicted ethane production rate, $F_{C2H6}^{\mathrm{model}}$ , at the optimized kinetic parameters. The best fit is achieved with $\Delta E_{ads-E}^{‡}$ = 42 kJ/mol, $\Delta E_{des-E}^{‡}$ = 38 kJ/mol, and *k*_eff_ = 10^12^ mol/m^2^/s, which correspond to the global minimum in χ^2^ , χ^2^_min_ = 3.6. Figure 5 shows that the values of *F*_C2H6_ predicted by the 2H’ mechanism for ethylene hydrogenation closely match the experimental measurements at low *P*_H2_ across most reaction temperatures; however, *F*_C2H6_ is underestimated at high *P*_H2_, especially as *T* increases. It is possible that this discrepancy can be accounted for by adding more complexity to the mechanism, for example, a second term in *r*_C2H6_ (eq 5) that includes the readsorption of ethane on the surface as the conversion increases beyond a certain threshold. Adding a readsorption term to eq 5 would add more flexibility to the 2H’ mechanism by allowing *F*_C2H6_ to remain low at low *P*_H2_ (through ethane readsorption) while increasing *F*_C2H6_ at high *P*_H2_ (through a decrease in $\Delta E_{ads-E}^{‡}$ , for example). The performance of the 2H’ mechanism might also be improved by allowing the pre-exponential factors for the rate constants parametrizing ethylene adsorption and desorption to vary around their transition state theory estimates. However, with an experimental data set consisting of only 28 points used for fitting, the addition of kinetic parameters for ethane readsorption and/or allowing the pre-exponential factors to vary would make the model incapable of converging on a unique solution. Nonetheless, the 2H’ mechanism for ethylene hydrogenation with only 3 degrees of freedom is able to reproduce the low conversion measurements of *F*_C2H6_ with reasonable accuracy. Therefore, the estimates obtained for $\Delta E_{ads-E}^{‡}$ , $\Delta E_{des-E}^{‡}$ , and *k*_eff_ are meaningful given the assumptions with which the 2H’ mechanism was derived.

**Figure 5:**
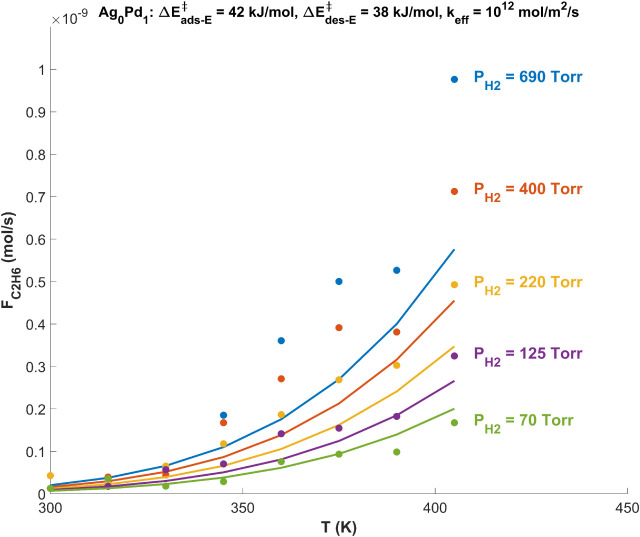
Flow rate of ethane, *F*_C2H6_ (mol/s), produced by the pure Pd catalyst (i.e., $x_{Pd}$ = 1, Ag_0_Pd_1_) versus the reaction temperature, *T* (K), at hydrogen pressures ranging from *P*_H2_ = 70 to 690 Torr. Experimental measurements of *F*_C2H6_ are shown by the data points, and the curves show the best fit solution of eq 9 to the data set with $\Delta E_{ads-E}^{‡}$ = 42 kJ/mol, $\Delta E_{\mathrm{des}-E}^{‡}$ = 38 kJ/mol, and *k*_eff_ = 10^12^ mol/m^2^/s. The values of *P*_H2_ at the right of the graph correspond to the data points and the fitted curve of the same color. $F_{C2H6}$ predicted by the 2H’ mechanism for ethylene hydrogenation closely matches the experimental measurements at low *P*_H2_ across nearly all reaction temperatures; however, *F*_C2H6_ is underestimated at high *P*_H2_, especially as T increases. It is possible that this discrepancy can be accounted for by adding more complexity to the model, namely a second term in *r*_C2H6_ that includes the readsorption of ethane on the surface as the conversion increases. Nonetheless, at low conversion the model-predicted *F*_C2H6_ is able to reproduce the experimental measurements with reasonable accuracy.

### 4.4 Evaluation of the Kinetic Parameters Predicted by the 2H’ Mechanism for Ethylene Hydrogenation

The kinetic parameters predicted by the 2H’ mechanism can be evaluated based on the agreement of the reaction conditions obtained at the best fit solution with experimental measurements. As explained in Section 3.1, the average ethylene hydrogenation reaction order in H_2_, *n*_H2_ = 0.69 ± 0.18 in the range *T* = 345–405 K, is consistent with the predictions of the 2H’ mechanism under reaction conditions where θ_H_ ≅ 1 and θ^’^_H_ ≅ 0 , with $n_{H2}=\frac{1}{2}$ , and conditions where θ_H_ ≅ 0 and θ^’^_H_ ≅ 1 , with *n* = 1 . Previously, we had proposed that $n_{H2}=\frac{1}{2}$ was the more likely scenario based upon the excess of H_2_ in the reactant mixture (i.e., *P*_H2_ *≫ P*_E_) and our expectations from the 2H’ mechanism for H_2_-D_2_ exchange, in which the subsurface was nearly vacant (i.e., θ^*’*^_H_ ≅ 0) .^22^ With estimates for $\Delta E_{\mathrm{ads}-E}^{‡}$ , $\Delta E_{\mathrm{des}-E}^{‡}$ , and *k*_eff_ obtained through fitting the experimental data set, all of the quantities parametrizing the 2H’ mechanism for ethylene hydrogenation are now known. Thus, the coverages of surface H (θ_H_) and subsurface H’ (θ^’^_H_) can be explicitly calculated to check these assumptions and the consistency of the model.

The kinetic parameters defining the 2H’ mechanism for ethylene hydrogenation $(v_{\mathrm{ads}}^{H2}$ , $v_{\mathrm{des}}^{H2}$ , $v_{ss}$ , $v_{ads}^{E}$ , $v_{\mathrm{des}}^{E}$ , $\Delta E_{\mathrm{ads}-H2}^{‡}$ , $\Delta E_{\mathrm{des}-H2}^{‡}$ , $\Delta E_{ss}$ , $\Delta E_{\mathrm{ads}-E}^{‡}$ , $\Delta E_{\mathrm{des}-E}^{‡}$ , and *k*_eff_) were used in the equations derived in the Supporting Information to find θ_H_ and θ^’^_H_ as a function of *P*_H2_ and *T*, as shown in Figure 6. The top row of subfigures in Figure 6 shows that at all 40 combinations of *P*_H2_ and *T* , applying the best-fit kinetic parameters results in a top surface that is H-saturated, i.e., θ_H_ ≅ 1, and a subsurface that is nearly vacant in H’, i.e., θ^*’*^_H_ ≅ 0. This is consistent with the coverages necessary for the 2H’ mechanism’s prediction of $n_{H2}=\frac{1}{2}$ . The bottom row of subfigures in Figure 6 shows the relative magnitude of the terms in the denominator of the rate equation for ethane production (eq 6), which were analyzed to obtain the predictions for *n*_H2_. At all *P*_H2_ and *T*, $\sqrt{K_{H2}P_{H2}}/\left(K_{E}P_{E}+1\right)\gg 1$ and $K_{ss}\sqrt{K_{H2}P_{H2}}\ll 0$ , which is consistent with the second column in Table 1 having θ_H_ ≅ 1 , θ*^’^*_H_ ≅ 0 , and $n_{H2}=\frac{1}{2}$ . Therefore, using the best-fit solution of the 2H’ mechanism to the experimental data results in a Pd surface that is highly saturated in H atoms, and consequently, highly depleted in adsorbed ethylene molecules (θ_E_ ≈ 0). This is consistent with the relatively strong interactions between H_2_ and Pd, which are expected to dominate the catalyst’s behavior, especially in H_2_-rich environments. The key takeaway from this analysis is that the values of $\Delta E_{\mathrm{ads}-E}^{‡}$ = 42 kJ/mol, $\Delta E_{\mathrm{des}-E}^{‡}$ = 38 kJ/mol, and *k*_eff_ = 10^12^ mol/m^2^/s found through kinetic parameter estimation on pure Pd yield surface H and subsurface H’ coverages that are consistent with the values of *n*_H2_ measured experimentally.

**Figure 6:**
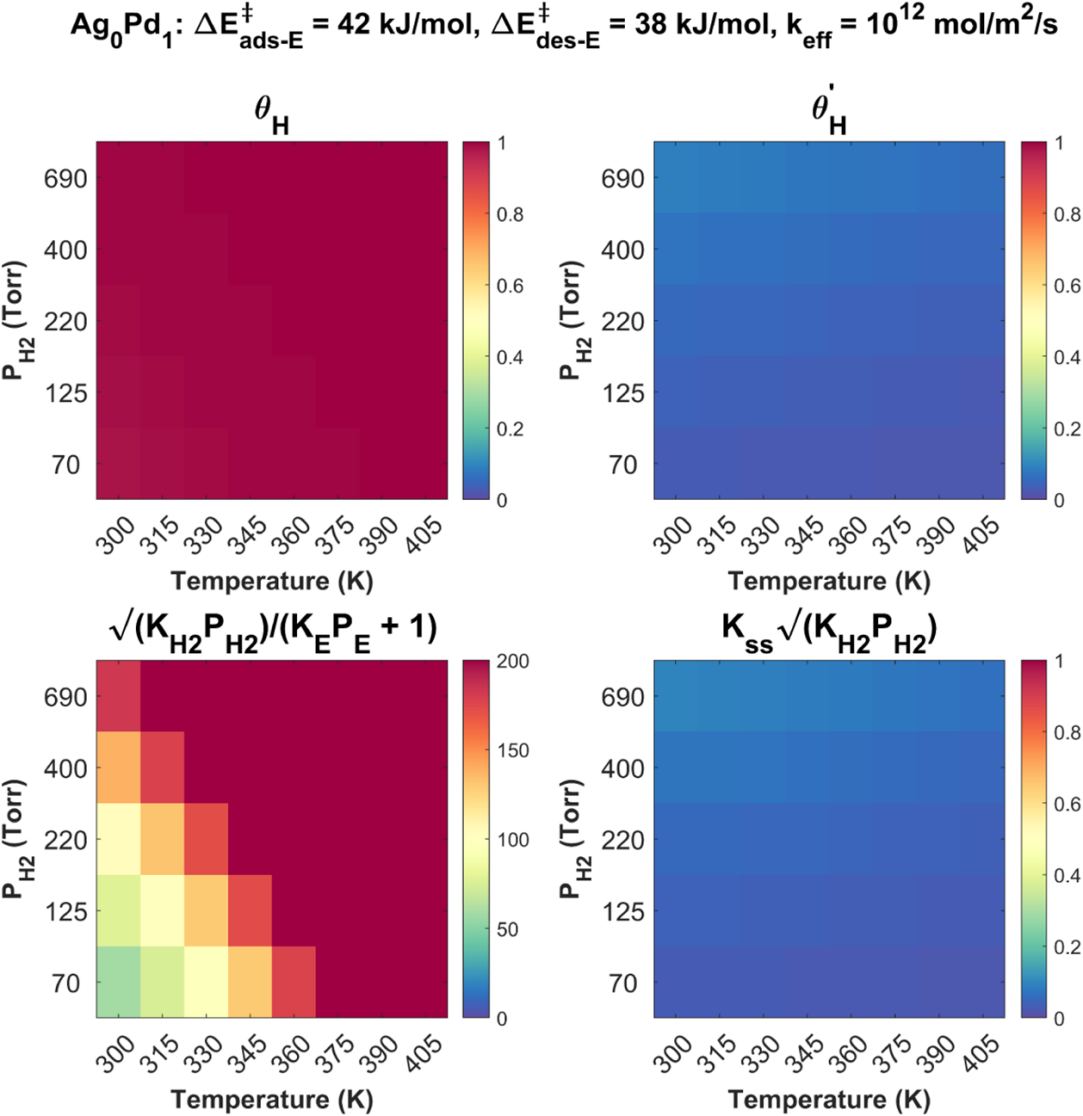
Coverages of H on the surface (θ_H_) and H’ in the subsurface (θ*^’^*_H_) (top row), and values of $\sqrt{K_{H2}P_{H2}}/\left(K_{E}P_{E}+1\right)$ and $K_{ss}\sqrt{K_{H2}P_{H2}}$ (bottom row) calculated using $\Delta E_{\mathrm{ads}-E}^{‡}$ = 42 kJ/mol, $\Delta E_{\mathrm{des}-E}^{‡}$ = 38 kJ/mol, and *k*_eff_ = 10^12^ mol/m^2^/s found from the best fit of eq 9 to the experimental data for Ag_0_Pd_1_ across all T and $P_{H2}$ . At all experimental conditions, the 2H’ mechanism for ethylene hydrogenation predicts θ_H_ ≅ 1 and *θ* ^’^_H_ ≅ 0, meaning that the surface is nearly saturated in H atoms and that the subsurface is nearly vacant in H’. In addition, $\sqrt{K_{H2}P_{H2}}/\left(K_{E}P_{E}+1\right)$ ≫ 1 by approximately 2 orders of magnitude, and $K_{ss}\sqrt{K_{H2}P_{H2}}≅$ 0. When $\sqrt{K_{H2}P_{H2}}/\left(K_{E}P_{E}+1\right)$ ≫1 and $K_{ss}\sqrt{K_{H2}P_{H2}}\ll 1$ , the rate law for ethane production (eq 6) predicts $n_{H2}=\frac{1}{2}$ with θ_H_ ≅ 1 and θ^’^_H_≅ 0 through simplification of the terms in the denominator. This prediction of $n_{H2}=\frac{1}{2}$ by the 2H’ mechanism is consistent with the experimentally measured values for *n*_H2_ shown in Figure 3, which predicts an overall average of *n*_H2_ = 0.69 ± 0.18 on Pd in the range *T* = 345–405 K.

The kinetic parameters predicted for ethylene hydrogenation can also be evaluated by comparing the fitted values of $\Delta E_{\mathrm{ads}-E}^{‡}$ , $\Delta E_{\mathrm{des}-E}^{‡}$ , and *k*_eff_ to expectations of ethylene adsorption and desorption onto/from Pd single crystal surfaces. Before this comparison can be made, however, it is important to quantify the uncertainty of the fitted kinetic parameters by visualizing the regions of parameter space that are capable of achieving a similar fit to the data as at the global minimum. In this way, we determine the level of confidence that can be obtained in the fitted solution. This is done by visualizing the hyper-ellipsoid encompassing the region of 95% confidence around the optimal parameter values $(\Delta E_{\mathrm{ads}-E}^{‡}$ = 42 kJ/mol, $\Delta E_{\mathrm{des}-E}^{‡}$ = 38 kJ/mol, log­(*k*_eff_) = 12) within $(\epsilon_{\mathrm{ads}-E}$ , $\epsilon_{\mathrm{des}-E}$ , and $\log\left(k_{\mathrm{eff}}\right))$ parameter space. This methodology was developed in our previous exploration of the kinetic parameters describing the H_2_-D_2_ exchange reaction using the 2H’ mechanism.^22,24^ In brief, the 95% confidence region around the fitted solution is constructed using the Hessian matrix returned by the solver, which is comprised of all of the second derivatives of the objective function (eq 9) with respect to the fitting parameters at the global minimum. The matrix of second derivatives showing the curvature of $\chi^{2}$ within parameter space allows construction of a 3D hyper-ellipsoid bounding the regions of $(\epsilon_{\mathrm{ads}-E}$ , $\epsilon_{\mathrm{des}-E}$ , and $\log\left(k_{\mathrm{eff}}\right))$ parameter space that are capable of producing equivalent fits to the data as the solution at χ^2^_min_ within the limit of 95% confidence. The 3D hyper-ellipsoid can be visualized in any 2D plane by taking the cross-section of the hyper-ellipsoid when the third parameter is fixed to its value at the global minimum, as in Figure 7. Figure 7 shows the three 2D contour plots of ln­(χ^2^) within $(\epsilon_{\mathrm{ads}-E}$ , $\epsilon_{\mathrm{des}-E}$ , and $\log\left(k_{\mathrm{eff}}\right))$ parameter space with the fitted solution marked by the blue dot at $(\Delta E_{\mathrm{ads}-E}^{‡}$ = 42 kJ/mol, $\Delta E_{\mathrm{des}-E}^{‡}$ = 38 kJ/mol, and log­(*k*_eff_) = 12) and the cross sections through the hyper-ellipsoid marked by the solid red error ellipses.

**Figure 7:**
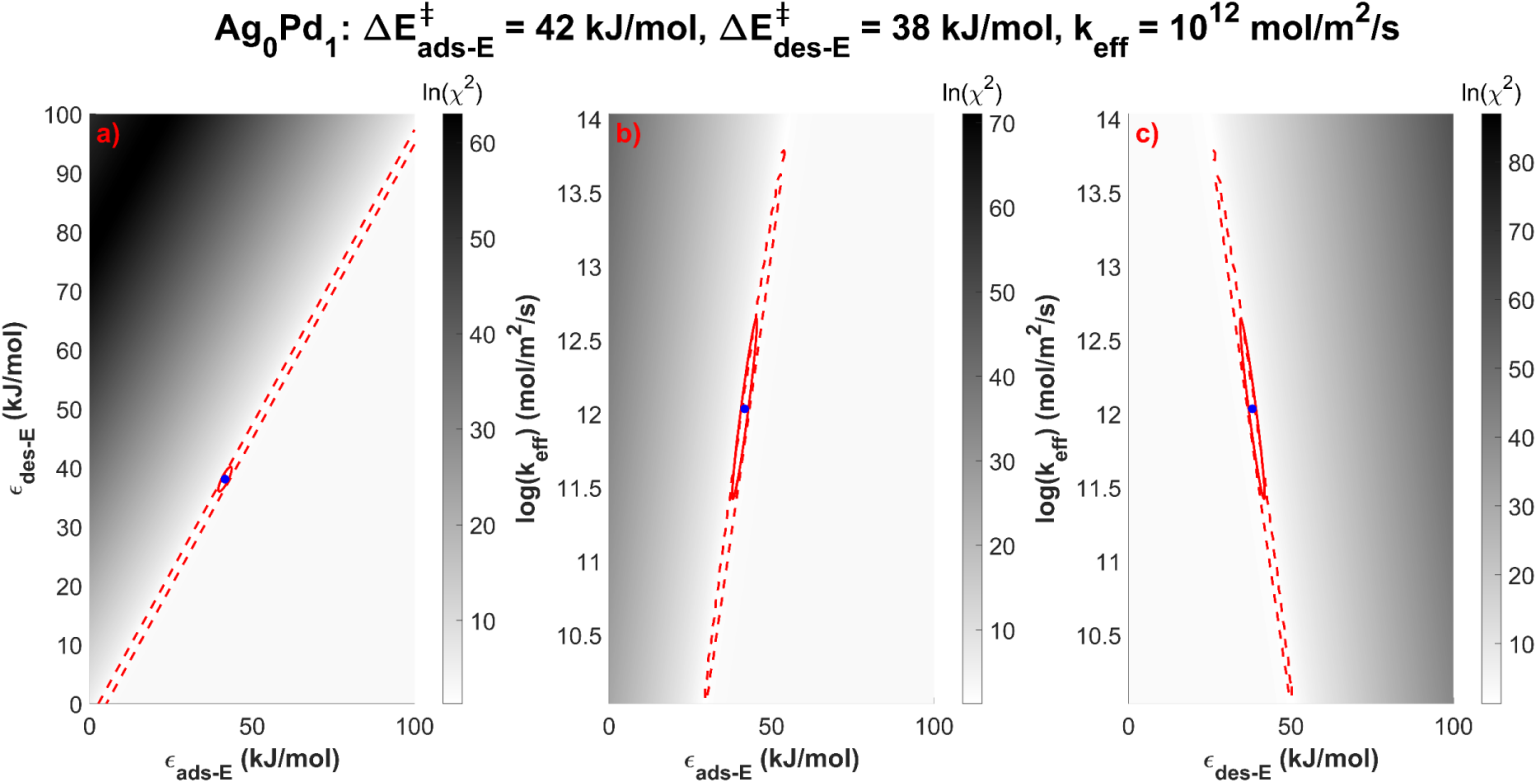
Grayscale contour plots of the sum of squared errors, log­(χ^2^) , within $(\epsilon_{\mathrm{ads}-E}$ , $\epsilon_{\mathrm{des}-E}$ , and $\log\left(k_{\mathrm{eff}}\right))$ parameter space when fitting the 2H’ mechanism for ethylene hydrogenation to the experimental data for the Ag_0_Pd_1_ catalyst. In all three plots, the blue dot marks the solution found by the solver at $\Delta E_{\mathrm{ads}-E}^{‡}$ = 42 kJ/mol, $\Delta E_{\mathrm{des}-E}^{‡}$ = 38 kJ/mol, and *k*_eff_ = 10^12^ mol/m^2^/s with χ^2^_min_ = 3.6. In each plot, one kinetic parameter is held constant at the value found by the solver while the other two are varied, i.e., (a) log­(*k*_eff_) = 12, (b) $\Delta E_{\mathrm{des}-E}^{‡}$ = 38 kJ/mol, and (c) $\Delta E_{\mathrm{ads}-E}^{‡}$ = 42 kJ/mol. The solid red error ellipses are 2D cross sections through the 3D hyper-ellipsoid that bounds the region of 95% confidence around the solution. The red dotted lines highlight the contour levels of constant ln­(χ^2^) found after performing a Taylor expansion from the solution found by the solver to any point on the solid red ellipses. The area bound by the dotted red lines serves as a conservative estimate for the 95% confidence interval within parameter space. In (b) and (c), the range log­(*k*_eff_) ≈ 10–14 yields solutions with the same quality of fit as at the global minimum. On the other hand, (a) shows strong coupling between $\epsilon_{\mathrm{ads}}$ and $\epsilon_{\mathrm{des}}$ to the point where an increase in one parameter can be compensated by a corresponding increase in the other parameter across the entire search space. In this case, the uncertainty of $\Delta E_{\mathrm{ads}-E}^{‡}$ and $\Delta E_{\mathrm{des}-E}^{‡}$ is large enough that the best-fit kinetic parameters do not sufficiently outperform the other possible solutions bounded by the red dotted lines. Nonetheless, (a) shows conclusively that all solutions have $\Delta E_{\mathrm{ads}-E}^{‡}>\Delta E_{\mathrm{des}-E}^{‡}$ since the region bounding the minima in ln­(χ^2^) lies underneath the line of parity $\epsilon_{\mathrm{des}-E}=\epsilon_{\mathrm{ads}-E}$ . This implies that the ethylene adsorption energy, i.e., $\Delta E_{\mathrm{ads}}^{E}$ = $\Delta E_{\mathrm{ads}-E}^{‡}-\Delta E_{\mathrm{des}-E}^{‡}$ , is always >0 and, therefore, slightly endothermic.

Since the contour plots of ln­(χ^2^) in Figure 7 exhibit nonquadratic behavior within parameter space around the global minimum, the overlaid red error ellipses cannot be directly used to define the regions of parameter space included in the 95% confidence region. Instead, a Taylor expansion can be performed from the global minimum at $\chi_{\min}^{2}$ to any point on the red ellipses to identify the constant contour level in χ^2^ that bounds the regions on the contour plot where all combinations of $(\epsilon_{\mathrm{ads}-E}$ , $\epsilon_{\mathrm{des}-E}$ , and $\log\left(k_{\mathrm{eff}}\right))$ produce an equivalent fit to the data within the limit of 95% confidence. In Figure 7, the red dotted lines surrounding the error ellipses mark the contour level at ln­(χ^2^) = 7.5 within which the landscape of χ^2^ does not change appreciably with $(\epsilon_{\mathrm{ads}-E}$ , $\epsilon_{\mathrm{des}-E}$ , and log­(*k*_eff_)). Inside this contour level lies narrow trenches in the plots of ln­(χ^2^) where the quality of the fit using different combinations of kinetic parameters is virtually indistinguishable. It is the extremes of these regions within parameter space that more accurately define the 95% confidence interval around $(\Delta E_{\mathrm{ads}-E}^{‡}$ , $\Delta E_{\mathrm{des}-E}^{‡}$ , log­(*k*_eff_)) found by the solver. From Figure 7b,c, the 95% confidence interval for *k*_eff_ ≈ 10^10^–10^14^ mol/m^2^/s, which is a symmetric range around the fitted solution, *k*_eff_ = 10^12^ mol/m^2^/s. An uncertainty range spanning 4 orders of magnitude for the apparent rate constant for ethylene hydrogenation is reasonable given that several mechanistic steps were combined in order to simplify the rate law for the 2H’ mechanism. On the other hand, Figure 7a shows that the uncertainty range for $\Delta E_{\mathrm{ads}-E}^{‡}$ and $\Delta E_{\mathrm{des}-E}^{‡}$ is very large, as it spans the entire parameter search space from 0 to 100 kJ/mol. In this case, the degree of coupling between $\Delta E_{\mathrm{ads}-E}^{‡}$ and $\Delta E_{\mathrm{des}-E}^{‡}$ in the 2H’ mechanism prevent us from accepting the solutions $\Delta E_{\mathrm{ads}-E}^{‡}$ = 42 kJ/mol and $\Delta E_{\mathrm{des}-E}^{‡}$ = 38 kJ/mol as the true global minimum. since other combinations of $\epsilon_{\mathrm{ads}-E}$ and $\epsilon_{\mathrm{des}-E}$ within the diagonal contour framed in Figure 7a can produce fits that are statistically equivalent to the solution shown in Figure 5. It is possible that more certainty around the optimized parameters could be achieved if the fitting had been performed over a data set that included a broader temperature and pressure range.

Despite the fact that the estimates for $\Delta E_{\mathrm{ads}-E}^{‡}$ and $\Delta E_{\mathrm{des}-E}^{‡}$ cannot be accepted as unique solutions, Figure 7a nonetheless reveals an important implication about the adsorption behavior of ethylene on Pd. In particular, the diagonal contour level indicating the region of $(\epsilon_{\mathrm{ads}-E}$ , $\epsilon_{\mathrm{des}-E})$ parameter space capable of fitting the data lies parallel to, but slightly below the line of parity, $\epsilon_{\mathrm{des}-E}$ = $\epsilon_{\mathrm{ads}-E}$ . This means that no matter where the true solution lies within this region, the parameters will always have $\Delta E_{\mathrm{ads}-E}^{‡}>\Delta E_{\mathrm{des}-E}^{‡}$ . In other words, the ethylene adsorption energy on Pd, defined as $\Delta E_{\mathrm{ads}}^{E}$ = $\Delta E_{\mathrm{ads}-E}^{‡}-\Delta E_{\mathrm{des}-E}^{‡}$ , is always >0 and therefore, slightly endothermic. Based on the values of $\Delta E_{\mathrm{ads}-E}^{‡}$ = 42 kJ/mol and $\Delta E_{\mathrm{des}-E}^{‡}$ = 38 kJ/mol found by the solver, it is expected that $\Delta E_{\mathrm{ads}}^{E}$ is on the order of ∼ 10 kJ/mol.

Density functional theory (DFT) investigations of ethylene adsorption on Pd surfaces have predicted $\Delta E_{\mathrm{ads}}^{E}$ as high as ∼ 90 kJ/mol depending on the geometry of the adsorption site and the tilt of the −CH_2_ groups with respect to the surface.^52,53^ However, DFT predictions of the ethylene adsorption energy decrease to as low as ∼ 30 kJ/mol when considering ethylene molecules adsorbed in the π-bonded configuration.^49,54^ It is worthwhile to note that whether ethylene is adsorbed in the di-σ-bonded configuration or in the π-bonded configuration, it does not influence our derivation of the 2H’ mechanism since both molecules occupy the same number of surface sites and have reaction pathways leading to the production of ethane.^18,20^ Changing from two di-σ-bonds occupying one surface site each to one π-bond occupying two surface sites would not change the equations for the model derived in the Supporting Information. The discrepancy in the ethylene adsorption energy can be reduced further since it has been shown that the interaction between H_2_ and ethylene is repulsive at short-range.^55^ Hence, at the high H_2_ coverage nearing saturation present in our experiments, the ethylene adsorption energy is expected to decrease below its DFT-calculated value in both adsorption modes. Thus, $\Delta E_{\mathrm{ads}}^{E}$ on the order of ∼10 kJ/mol is a reasonable approximation, especially since the presence of subsurface H’ in Pd-rich alloys possibly destabilizes ethylene adsorption even further.

## 5 Conclusion

In this work, we extended the 2H’ framework established for H_2_-D_2_ exchange to obtain a microkinetic model for ethylene hydrogenation on Ag_*x*_Pd_1–*x*_ alloy catalysts that incorporates the presence of subsurface hydrogen, H’, into the reaction mechanism. The traditional Horiuti-Polanyi mechanism was simplified and adapted in a way that allowed us to preserve the molecular adsorption of ethylene and dissociative adsorption of H_2_ while incorporating the effect of subsurface H’ that is necessary to activate surface H atoms and facilitate ethylene hydrogenation. We derived a rate law for ethylene hydrogenation using the 2H’ framework and compared the implications of the proposed mechanism with experimental measurements of ethylene hydrogenation taken across a Ag_*x*_Pd_1–*x*_ CSAF. The 2H’ mechanism for ethylene hydrogenation on Pd was consistent with the average reaction order in H_2_ measured experimentally, $n_{H2}$ = 0.69 ± 0.18. The 2H’ mechanism’s prediction of $n_{H2}=\frac{1}{2}$ under conditions of high H surface coverage (θ_H_ ≅ 1) and low H’ subsurface coverage $\left(\theta_{H}^{\prime }≅0\right)$ closely matches the reaction order obtained using the Ag_*x*_Pd_1–*x*_ CSAF, especially at low reaction temperatures, e.g., $n_{H2}$ = 0.53 at 345 K. Finally, the ethane production rate given by the 2H’ mechanism was fit to the experimentally measured ethane production rate on the pure Pd catalyst to estimate the remaining kinetic parameters defining the reaction mechanism. Kinetic parameter estimation bounded the effective hydrogenation rate constant, *k*_eff_, to between 10^10^ and 10^14^ mol/m^2^/sec and predicted an endothermic ethylene adsorption energy $\left(\Delta E_{\mathrm{ads}}^{E}\right)$ on the order of ∼10 kJ/mol. Kinetic parameter estimates confirm that the surface H coverage (θ_H_) and subsurface H’ coverage (θ^*’*^_H_) predicted by the model are consistent with the experimental conditions necessary to obtain the measured values of *n*_H2_. The consistency of the 2H’ mechanism with experimental measurements of ethylene hydrogenation on our Ag_*x*_Pd_1–*x*_ CSAF shows the potential for including the influence of subsurface hydrogen in modeling increasingly complex surface reactions.

## Supplementary Material



## Notes

Linear fits of log­(ξ) versus log­(*P*_H2_) to estimate *n*_H2_ versus Ag_*x*_Pd_1–*x*_ alloy composition. Derivation of the 2H’ mechanism for ethylene hydrogenation­(PDF)
