# Supplementary material for: The Dual Subsurface Hydrogen (2H’) Mechanism for Ethylene Hydrogenation on Pd
Source: J Phys Chem C Nanomater Interfaces. 2025 Aug 7;129(33):14747–60. doi: 10.1021/acs.jpcc.5c02240 (PMC12376113; doi:10.1021/acs.jpcc.5c02240)
Supplement: Supplementary file 1 [file jp5c02240_si_001.pdf]

Supporting Information

The Dual Subsurface Hydrogen (2H') Mechanism for Ethylene Hydrogenation on Pd

Nicholas Golio[a] and Andrew J Gellman[a,b*]
[a]Department of Chemical Engineering
Carnegie Mellon University
5000 Forbes Ave., Pittsburgh, PA 15213, United States
[b]W.E. Scott Institute for Energy Innovation
Carnegie Mellon University
5000 Forbes Ave., Pittsburgh, PA 15213, United States

*Corresponding author: gellman@cmu.edu, +1 (412) 268-3848

1. Linear fits of $\log(\xi)$ versus $\log(P_{H2})$ to estimate n_{H2} versus Ag_xPd_{1-x} alloy composition

Figure S1 shows the plots of $\log(\xi)$ versus $\log(P_{H2})$ for the 14 most Pd-rich catalysts (several of which have nominally identical bulk compositions) ranging from x_{Pd} = 1 → 0.93. Since Ag_xPd_{1-x} catalysts with $x_{Pd} \leq 0.90$ were inactive for ethylene hydrogenation, Figure S1 contains all of the estimates for n_{H2} at low conversion (i.e., $\xi < 0.1$) that can be obtained from this data set. For these data points with $\xi < 0.1$, the reaction is considered to be at low conversion and this allows us to neglect the re-adsorption of ethane molecules from the gas phase onto the surface. Note that data points with $\xi < 0.02$ (i.e., $\log(\xi) < -1.7$) were excluded from the analysis due to their conversion falling below the noise level. In Figure S1, all datapoints of the same color were measured at the same reaction temperature and are fitted by a line of best fit, the slope of which yields n_{H2}. The values of n_{H2} estimated for different catalysts with the same bulk alloy composition exhibit a high degree of internal consistency within this data set over an order of magnitude change in P_{H2}. Averaging across all reaction temperatures and catalyst compositions yields $n_{H2} = 0.69 \pm 0.18$ for ethylene hydrogenation on Pd within the range T = 345 K → 405 K.

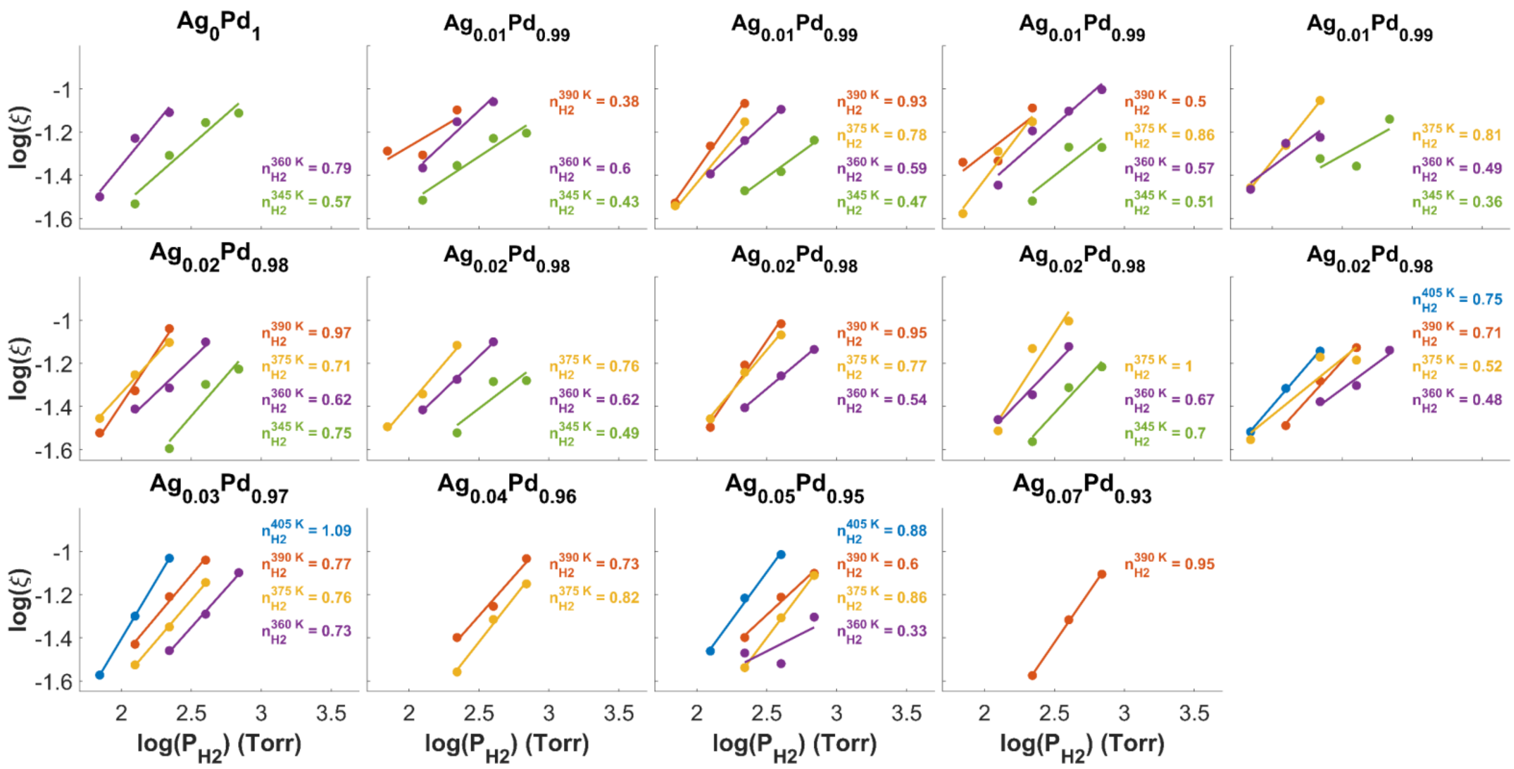

Figure S1. Logarithm of ethylene conversion, $\log(\xi)$, versus logarithm of P_{H2}, $\log(P_{H2})$, for Ag_xPd_{1-x} catalysts with $0.93 \leq x_{Pd} \leq 1$ when T = 345 K → 405 K. The relationship between $\log(\xi)$ and $\log(P_{H2})$ was fit using a line, the slope of which estimates the reaction order with respect to hydrogen, n_{H2}, which is printed on the right side of each subplot in the same color as the linear fit that it describes. Note that conversion measurements below the noise level (i.e., ξ < 0.02 or $\log(\xi)$ < -1.7) were excluded from the plots and from the analysis. Reaction data at low T and catalysts with x_{Pd} < 0.93 were not sufficiently active for ethylene hydrogenation and therefore, this figure contains all possible values of n_{H2} that can be estimated using the low conversion data set. Several subplots show different catalysts with the same nominal bulk composition, which have good agreement in n_{H2} at all reaction temperatures, indicating that the results are internally consistent. The minimum value of n_{H2} = 0.33 occurs on $Ag_{0.05}Pd_{0.95}$ at 360 K and the maximum value of n_{H2} = 1.09 occurs on $Ag_{0.03}Pd_{0.97}$ at 405 K. The average reaction order in H_2 across the entire data set is n_{H2} = 0.69 ± 0.18 within the range T = 345 K → 405 K.

2. Ethylene hydrogenation kinetics via the Dual Subsurface Hydrogen (2H') mechanism

The kinetics of ethylene hydrogenation can be described by the 2H' mechanism by extending the framework that was previously established for the H_2-D_2 exchange reaction. According to the 2H' mechanism, H_2 adsorption and desorption on the top surface is facilitated by the presence of two adjacent H' in the immediate subsurface. Similarly, the presence of H' can influence the hydrogenation of adsorbed ethylene molecules (E) by interacting with surface H in their vicinity, causing them to react with ethylene. As for H_2 adsorption and desorption, ethylene hydrogenation requires two H' in the subsurface to interact with two H atoms on the top surface in order to fully hydrogenate the ethylene molecule. In the case of ethylene hydrogenation, however, the two H' need not be adjacent (as for H_2 adsorption and desorption) as long as there is one H' present in the corresponding subsurface for each H participating in the hydrogenation. As for the 2H' mechanism for H_2-D_2 exchange, the exchange of H between the surface and the subsurface is determined by the diffusion equilibrium constant, K_{ss}. The elementary steps for the 2H' mechanism for ethylene hydrogenation are shown below with the rate constants describing the forward and reverse reactions printed to the right.

$$H_2(g) + 2* \underset{2H'}{\longleftrightarrow} 2H* \qquad k_{ads}^{H2}, k_{des}^{H2}$$

$$E(g) + ** \leftrightarrow E** \qquad k_{ads}^{E}, k_{des}^{E}$$

$$H* + *' \leftrightarrow H*' + * \qquad k_{ss}^{in}, k_{ss}^{out}$$

$$2H* + E** \underset{2H'}{\longrightarrow} HEH(g) + 4* \qquad k_{eff}$$

In the mechanism for ethylene hydrogenation provided above, $*$ denotes an empty surface site, $H*$ and $E*$ represent adsorbed H atoms and ethylene molecules, respectively, and the superscript $'$ indicates that the corresponding species is in the subsurface. Note that although the adsorption of both $H_2(g)$ and $E(g)$ require two adjacent surface sites, a different notation is used for each adsorption process since H_2 adsorbs dissociatively and ethylene adsorbs molecularly. The $2H'$ symbol beneath certain reaction arrows represent the presence of two subsurface H' which activate the corresponding surface sites for H_2 adsorption and ethylene hydrogenation. Surface-to-subsurface diffusion of H atoms is given by the equilibrium constant $K_{ss} = k_{in}^{ss}/k_{out}^{ss}$. In the limit of low conversion, the rate of ethane production given by the 2H' mechanism is $r_{C2H6} = k_{eff}\theta_E\theta_H^2{\theta'_H}^2$, where k_{eff} is the effective hydrogenation rate constant (mol/m^2/sec) and θ_E, θ_H, and θ'_H represent the fractional coverages of ethylene (E), H, and H', respectively. It is important to note that the equation for ethylene hydrogenation, as written, does not necessarily require that both hydrogenation steps occur concurrent with the molecular desorption of ethane into the gas phase. Rather, the expression approximates the rate limiting step of ethylene hydrogenation (often believed to be the addition of the first H atom) with the assumption that the other steps occur relatively quickly and are thus, not kinetically relevant.

Equations for the 2H' mechanism

Variables: θ_H, θ_E, θ'_H

Rate and equilibrium constants: $K_{H2} = k_{ads}^{H2}/k_{des}^{H2}$, $K_E = k_{ads}^{E}/k_{des}^{E}$, $K_{ss} = k_{ss}^{in}/k_{ss}^{out}$, k_{eff}

Pressures: P_{H2}, P_E, P_{HEH}

Steady-state and mass balance equations:

$$\frac{d\theta_H}{dt} = 0 = 2k_{ads}^{H2}P_{H2}\left(1-\theta_H-\frac{1}{2}\theta_E\right)^2 {\theta'_H}^2 - 2k_{des}^{H2}\theta_H^2{\theta'_H}^2 - k_{ss}^{in}\theta_H(1-\theta'_H)$$

$$+ k_{ss}^{out}\theta'_H\left(1-\theta_H-\frac{1}{2}\theta_E\right) \qquad (1)$$

$$\frac{d\theta_E}{dt} = 0 = k_{ads}^{E}P_E\left(1-\theta_H-\frac{1}{2}\theta_E\right) - \frac{1}{2}k_{des}^{E}\theta_E \qquad (2)$$

$$\frac{d\theta'_H}{dt} = 0 = k_{ss}^{in}\theta_H(1-\theta'_H) - k_{ss}^{out}\theta'_H\left(1-\theta_H-\frac{1}{2}\theta_E\right) \qquad (3)$$

Solution of the 2H' mechanism

Eq. 2 →

$$\theta_E = \frac{2K_EP_E(1-\theta_H)}{1+K_EP_E}$$

Eq. 3 →

$$\theta'_H = \frac{K_{ss}\theta_H}{1-\theta_H-\frac{1}{2}\theta_E+K_{ss}\theta_H}$$

Eq. 1 using θ_E and θ'_H →

$$\theta_H = \frac{\sqrt{K_{H2}P_{H2}}}{1+\sqrt{K_{H2}P_{H2}}+K_EP_E} \qquad (4)$$

Eqs. 2 & 4 →

$$\theta_E = \frac{2K_E P_E}{1 + \sqrt{K_{H2} P_{H2}} + K_E P_E} \tag{5}$$

Eqs. 3 & 4 →

$$\theta_H' = \frac{K_{ss}\sqrt{K_{H2} P_{H2}}}{1 + K_{ss}\sqrt{K_{H2} P_{H2}}} \tag{6}$$

Summary of coverages for the 2H' mechanism

$$\theta_H = \frac{\sqrt{K_{H2} P_{H2}}}{1 + \sqrt{K_{H2} P_{H2}} + K_E P_E} \tag{4}$$

$$\theta_E = \frac{2K_E P_E}{1 + \sqrt{K_{H2} P_{H2}} + K_E P_E} \tag{5}$$

$$\theta_H' = \frac{K_{ss}\sqrt{K_{H2} P_{H2}}}{1 + K_{ss}\sqrt{K_{H2} P_{H2}}} \tag{6}$$

Ethane production rate at low conversion (i.e., $P_{HEH} \approx 0$)

The rate law for ethane production, r_{C2H6}, is given by equation 7. The expressions for θ_H, θ_E, and θ_H' can be substituted into the rate law to yield an expression for the ethane production rate in terms of rate and equilibrium constants (K_{H2}, K_E, K_{ss}, and k_{eff}) and experimental parameters (P_{H2} and P_E).

$$r_{C2H6} = \frac{1}{2}k_{eff}\theta_E\theta_H^2{\theta'_H}^2 = \frac{k_{eff}K_{ss}^2K_EP_EK_{H2}^2P_{H2}^2}{\left(1+\sqrt{K_{H2}P_{H2}}+K_EP_E\right)^3\left(1+K_{ss}\sqrt{K_{H2}P_{H2}}\right)^2} \quad (7)$$

Reaction order in P_{H2}

The reaction order with respect to hydrogen, n_{H2}, describes the dependence of the ethane production rate on the hydrogen partial pressure, P_{H2}. The rate law for ethane production in eq. 7 can be simplified based upon which terms in the denominator are expected to be dominant. When $\theta'_H \cong 1$ and $\theta_H \cong 1$, it results in conditions where $K_{ss}\sqrt{K_{H2}P_{H2}} \gg 1$ and $\sqrt{K_{H2}P_{H2}}/(K_EP_E+1) \gg 1$ (i.e., $\sqrt{K_{H2}P_{H2}} \gg (1+K_EP_E)$), which allows the H_2 pressure dependence in eq. 7 to be simplified to $r_{C2H6} \sim P_{H2}^{-1/2}$, resulting in $n_{H2} = -\frac{1}{2}$. When $\theta'_H \cong 0$ and $\theta_H \cong 1$, $K_{ss}\sqrt{K_{H2}P_{H2}} \ll 1$ and $\sqrt{K_{H2}P_{H2}}/(K_EP_E+1) \gg 1$, which results in $n_{H2} = \frac{1}{2}$. When $\theta'_H \cong 1$ and $\theta_H \cong 0$, $K_{ss}\sqrt{K_{H2}P_{H2}} \gg 1$ and $\sqrt{K_{H2}P_{H2}}/(K_EP_E+1) \ll 1$, which results in $n_{H2} = 1$. And finally, when $\theta'_H \cong 0$ and $\theta_H \cong 0$, $K_{ss}\sqrt{K_{H2}P_{H2}} \ll 1$ and $\sqrt{K_{H2}P_{H2}}/(K_EP_E+1) \ll 1$, which results in $n_{H2} = 2$. The simplification of eq. 7 to obtain n_{H2} for each set of conditions is shown below:

$$K_{ss}\sqrt{K_{H2}P_{H2}} \gg 1 \text{ and } \sqrt{K_{H2}P_{H2}}/(1+K_EP_E) \gg 1: \quad r_{C2H6} \sim \frac{P_{H2}^2}{P_{H2}^{3/2}P_{H2}} \sim P_{H2}^{-1/2} \qquad n_{H2} = -\frac{1}{2}$$

$$K_{ss}\sqrt{K_{H2}P_{H2}} \ll 1 \text{ and } \sqrt{K_{H2}P_{H2}}/(1+K_EP_E) \gg 1: \quad r_{C2H6} \sim \frac{P_{H2}^2}{P_{H2}^{3/2}} \sim P_{H2}^{1/2} \qquad n_{H2} = \frac{1}{2}$$

$$K_{ss}\sqrt{K_{H2}P_{H2}} \gg 1 \text{ and } \sqrt{K_{H2}P_{H2}}/(1+K_EP_E) \ll 1: \quad r_{C2H6} \sim \frac{P_{H2}^2}{P_{H2}} \sim P_{H2} \qquad n_{H2} = 1$$

$K_{ss}\sqrt{K_{H2}P_{H2}} \ll 1$ and $\sqrt{K_{H2}P_{H2}}/(1 + K_E P_E) \ll 1$: $\quad r_{C2H6} \sim P_{H2}^2 \quad n_{H2} = 2$
